# HGF/MET Axis Induces Tumor Secretion of Tenascin-C and Promotes Stromal Rewiring in Pancreatic Cancer

**DOI:** 10.3390/cancers13143519

**Published:** 2021-07-14

**Authors:** Chiara Modica, Martina Olivero, Francesca Zuppini, Melissa Milan, Cristina Basilico, Elisa Vigna

**Affiliations:** 1 Candiolo Cancer Institute, FPO-IRCCS, 10060 Candiolo, Italy; modicachiara89@gmail.com (C.M.); martina.olivero@ircc.it (M.O.); francesca.zuppini@unito.it (F.Z.); melissa.milan@ircc.it (M.M.); 2 Department of Oncology, University of Turin, 10060 Candiolo, Italy

**Keywords:** pancreatic ductal adenocarcinoma, tumor microenvironment, hepatocyte growth factor, MET oncogene, tenascin C, metastasis

## Abstract

**Simple Summary:**

It has been previously shown that activation of the MET receptor by its ligand, the hepatocyte growth factor (HGF), modulates the tumor-stroma cross-talk in models of pancreatic cancer. We now wish to cast light on the molecular mechanisms by which this ligand/receptor pair sustains the interaction between cancer cells and the tumor microenviroment. To this end, we compared data obtained by large-scale analysis of gene expression in pancreatic cancer cells grown in the presence of HGF versus cells grown in the presence of HGF and treated with specific inhibitors of HGF/MET signaling. By clustering differentially expressed genes according to functional groups, we identified candidate genes involved in the process. Among these, tenascin C was selected due to its activity in sustaining the malignant phenotype. Our results highlight a new role for tenascin C, which could represent the operative arm through which MET promotes activation of the stromal compartment in pancreatic cancer.

**Abstract:**

Pancreatic ductal adenocarcinoma is an aggressive tumor characterized by the presence of an abundant stromal compartment contributing significantly to the malignant phenotype. Pancreatic stellate cells are peculiar fibroblasts present in the stroma and represent the predominant source of extracellular matrix proteins, pro-inflammatory cytokines, and growth factors, including hepatocyte growth factor (HGF). Exploiting a co-culture system of human pancreatic stellate cells and cancer cells, we demonstrated that fibroblast activation was reduced upon HGF/MET axis inhibition. To unveil the signaling pathways sustaining stroma modulation orchestrated by MET activation in the tumor, we analyzed the gene expression profile in pancreatic cancer cells stimulated with HGF and treated with HGF/MET inhibitors. Transcriptome analysis showed that, among all the genes modulated by HGF, a subset of 125 genes was restored to the basal level following treatment with the inhibitors. By examining these genes via ingenuity pathway analysis, tenascin C emerged as a promising candidate linking MET signaling and tumor microenvironment. MET-dependent tenascin C modulation in pancreatic cancer cells was validated at RNA and protein levels both in vitro and in vivo. In conclusion, this work identifies tenascin C as a gene modulated by MET activation, suggesting a role in MET-mediated tumor-stroma interplay occurring during pancreatic tumor progression.

## 1. Introduction

Pancreatic ductal adenocarcinoma (PDAC) is the most common type of pancreatic cancer and is characterized by highly aggressive behavior [1]. According to the PDAC progression model, invasive adenocarcinoma arises from a well-defined duct lesion, called a pancreatic intraepithelial neoplasia, which evolves into a malignant adenocarcinoma through the accumulation of molecular abnormalities [2,3]. One of the hallmarks of PDAC lesions is the presence of an extensive fibrotic tissue deposition around tumor cells, termed desmoplasia [4]. The non-cellular components of desmoplasia incorporate multiple extracellular matrix proteins such as collagen, laminin, fibronectin, proteoglycans, glycoproteins, and polysaccharides. Desmoplasia is also characterized by the presence of miscellaneous cellular components including a variety of stromal cell types, such as fibroblasts, endothelial cells, and pericytes, as well as a number of infiltrating immune cell types [5]. These cells, recruited and forced by cancer cells to secrete cytokines, chemokines, growth factors, extracellular matrix proteins, and remodeling enzymes, promote the development of a peculiar microenvironment. This compartment strongly supports tumor growth, fosters metastasis dissemination, and limits drug penetration and uptake, thus significantly contributing to the maintenance and intensification of the malignant phenotype [6]. Indeed, pancreatic cancer cells feature a ‘non-cell autonomous’ behavior, establishing complex and bidirectional paracrine loops with the surrounding microenvironment [7]. Among the cellular components of the PDAC stroma, pancreatic stellate cells (PSCs) are the predominant source of extracellular matrix proteins, pro-inflammatory cytokines, and growth factors [8,9]. In physiological conditions, PSCs are rare and quiescent cells, characterized by the presence of intracellular lipid droplets and the expression of glial fibrillary acidic protein, vimentin, nestin, and desmin [10]. In inflammatory or stress conditions, quiescent PSCs acquire a myofibroblast-like phenotype characterized by morphological changes such as: (i) acquisition of a spindle-shaped morphology, (ii) loss of lipid vacuoles, (iii) expression of the activation marker protein α-smooth-muscle actin (α-SMA), and (iv) increased proliferation and secretion of extracellular matrix components [11]. Moreover, activated PSCs produce high levels of many different growth factors and cytokines, acting, on one hand, on themselves, sustaining their own active state, and, on the other hand, on cancer cells, supporting their growth and the invasive phenotype [12,13]. One of these factors is hepatocyte growth factor (HGF) [14], the high-affinity ligand of MET.

HGF-sustained MET-activation in cancer cells exacerbates their aggressiveness, prompting dissemination from the primary tumor to regional and distant sites [15,16]. Interestingly, administration of an HGF-targeting antibody (AMG-102) [17] in experimental models of pancreatic cancer was able to produce a phenotypic reprogramming of stromal cells that ultimately led to decreased collagen deposition [18]. This result indicates that the paracrine stroma/cancer signaling through the HGF/MET pathway not only sustains the malignant phenotype of transformed cells, but also promotes the activation of the stromal compartment. However, the molecular mechanisms underlining this process are still unknown. We previously demonstrated that the combination of a MET-targeting antibody (MvDN30) with an HGF-sequestering protein (DecoyMET^K842E^) strongly inhibits the HGF/MET axis in HPAF-II cells, resulting in a dramatic reduction in the ability of pancreatic cancer cells to metastasize to the lungs [19,20]. In the present study, we exploited the previously established dual antibody/decoy inhibitory strategy to investigate the molecular pathways modulated by HGF/MET inhibition in pancreatic cancer cells, and identify tenascin C (TNC) as one of the factors secreted by cancer cells that could sustain the stromal rewiring of pancreatic cancers.

## 2. Materials and Methods

### 2.1. Cell Culture

HPAF-II and Capan-1 human pancreatic adenocarcinoma cells were obtained from ATCC/LGC Standards S.r.l. (Sesto San Giovanni, Italy), and cultured as suggested by the supplier. Human pancreatic stellate cells (PSCs) were purchased from ScienCell Research Laboratory (Carlsbad, CA, USA) and cultured in poly-L-lysine-coated flasks as recommended. All cell lines were routinely tested for mycoplasma.

### 2.2. Co-Culture Models

For the direct co-culture model, 3 × 10^5^ HPAF-II or Capan-1 cells were plated in poly-L-lysine-coated 6-well plates together with 1 × 10^5^ PSCs in Stellate Cell Medium (ScienCell Research Laboratory). For Combo treatment, MvDN30 (1 µM) and DecoyMET^K842E^ (1 µM) (produced and purified by U-Protein Express BV, Utrech, The Netherlands) were added. Medium was partially removed and replaced with fresh medium plus inhibitors every two days. After 5 days, cells underwent immunofluorescence analysis. For the indirect co-culture model, HPAF-II or Capan-1 cells (1 × 10^5^/well) were seeded in the upper chamber of 24-transwell plates (pore size 0.4 μm; BD Falcon, Bedford, MA, USA), while PSCs (7 × 10^4^/well) were seeded in the lower chamber on poly-L-lysine-coated wells. Cells were cultured and treated as above. After 5 days of co-culture, RNA was extracted from PSC population and analyzed by quantitative real-time polymerase chain reaction (qRT-PCR).

### 2.3. Western Blot Analysis

For evaluation of the TNC expression in the supernatants, HPAF-II cells were stimulated with HGF (20 ng/mL) and treated or not with Combo for 5 days. Then, equal amounts of conditioned cell medium were resolved by SDS-PAGE, and transferred to 0.2 μm nitrocellulose Trans-Blot Turbo TM membranes (Thermo Fisher Scientific, Whaltman, MA, USA). For protein detection, the tenascin C antibody D16C4 (Cell Signaling Technology, Beverly, MA, USA) was used. Western blot bands were quantified with ImageJ software.

### 2.4. RNA Analysis and Real-Time PCR

Total RNA from cancer cells, treated or not with Combo for 24 h and then stimulated with HGF (20 ng/mL) for 6 h, was obtained using ^®^ RSC simplyRNA Tissue Kit (Promega Corp, Madison, WI, USA) following manufacturer’s instructions. For gene expression array, 1 µg of total RNA was retro-transcribed with iScript^tm^ cDNA Synthesis Kit (Hercules, CA, USA) and qRT-PCR was performed using the following Taqman probes from Applied Biosystem: α-SMA (Hs00909449_m1), FAP (Hs00990806_m1), and TNC (Hs01115665_m1). GAPDH (Hs99999905_m1) were used as housekeeping gene to normalize mRNA levels, and data were analyzed using the comparative CT method (ΔΔCt analysis). For RNAseq analysis, total RNA was extracted from HPAF-II cells treated as above, using Maxwell^®^ RSC simplyRNA Tissue Kit (Promega Corp) according to the manufacturer’s instructions. Agilent 2100 Bioanalyzer (Agilent Technologies, Palo Alto, CA, USA) was used to evaluate RNA integrity.

RNA-seq libraries were generated using TruSeq RNA Sample Prep Kit v2 (Illumina, San Diego, CA, USA) according to manufacturer’s recommendations. Differential gene expression analysis was generated using the DESeq2 package [21], embedded in SeqBox [22], using as thresholds adjusted *p*-value < 0.1 and absolute log2 fold change ≥1 and ≤−1. Hierarchical clustering was obtained applying the following parameters: Euclidean distance and average linkage. Ingenuity Pathway Analysis (Qiagen, Hilden, Germany) was done on the identified differentially expressed genes.

### 2.5. Immunofluorescence

Immunofluorescence analysis on tumor cells and tissues was performed as described [23,24]. Staining was carried out with the following primary antibodies: anti-αSMA (ab5694), anti-TNC (ab108930), and anti-Collagen-I (ab34710) all from Abcam (Cambridge, UK), and revealed by Alexa Fluor 488-conjugated secondary antibody. All images were captured with a Leica TCS SP5 AOBS confocal laser-scanning microscope (Leica Microsystems, Wetzlar, Germany). Immunofluorescence acquisition settings were kept constant within each cell line or tumor tissue. Mean fluorescence intensity (MFI) was evaluated with ImageJ software, measuring the mean pixel intensity in each channel, background subtracted. MFI was normalized on DAPI tumors.

### 2.6. In Vivo Experiments

In vivo experiments were performed according to protocols approved by the Ethical Committee for animal experimentation of the Candiolo Cancer Institute and by the Italian Ministry of Health. hHGF-ki SCID mice were obtained from AVEO Pharmaceuticals, (Cambridge, MA, USA). Capan-1 or HPAF-II cells were transduced with 100 ng/mL p24 of lentiviral vectors for expression of the luciferase gene [25] as described [26]. Luciferase-expressing cells (10^5^ cells/mouse in 50 μL of PBS) were injected in the pancreas of 4- to 6-week-old female hHGF-ki SCID mice. After 3 days, XenoLight D-Luciferin (150 mg/kg; Perkin Elmer, Waltham, MA, USA) was injected intraperitoneally into the mice. Based on the bioluminescence signal measured by an IVIS Spectrum CT apparatus (Perkin Elmer), mice were stratified into homogeneous groups and randomly assigned to the treatment arms. Vehicle (PBS), MvDN30 (10 mg/kg, daily), or Combo treatment (MvDN30, 10 mg/kg daily; decoyMET^K842E^, 10 mg/kg every two days) were administered by intraperitoneal injection. At sacrifice, tumors were excised and analyzed by immunofluorescence.

### 2.7. Generation of TNC-shRNA HPAF-II Cells

HPAF-II cells were plated in 6-well costar dishes (10^5^ cells/well) and infected for 24 h with lentiviral vector particles expressing TNC shRNAs (NM_002160 from Sigma Aldrich, St. Louis, MO, USA) in the presence of 8 μg/mL polybrene. Two different particle clones (TRNC0000154001 and TRNC0000157688) were mixed, for a total of 4 × 10^5^ vector particles/well. Efficacy of TNC silencing was checked by qRT-PCR performed as described above on mRNA extracted from cells treated or not with 20 ng/mL of HGF for 6 h.

### 2.8. Statistical Analysis

Average, standard deviation (SD), and standard error of the mean (SEM) were calculated using Microsoft Office Excel 2010 software (Microsoft Corporation, Redmond, WA, USA) or GraphPad Prism Software. Statistical significance was determined using the two-tailed Student’s *t* test or Mann–Whitney test. A value of *p* ≤ 0.05 was considered significant.

## 3. Results

### 3.1. Inhibition of the HGF/MET Axis Reduces Activation of Pancreatic Stellate Cells

**Figure 1:**
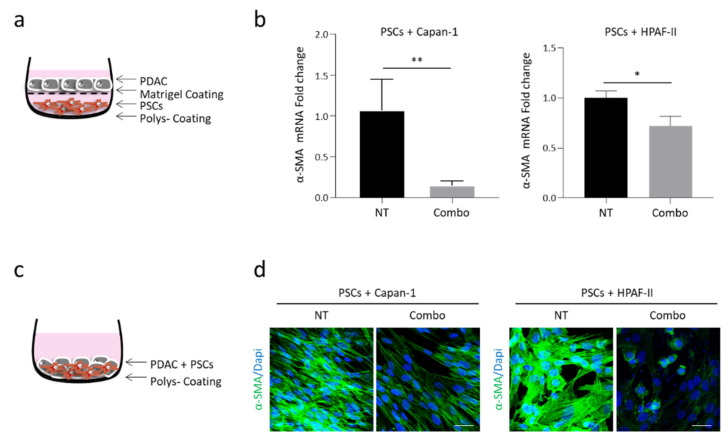
Concomitant HGF/MET inhibition reduces α-SMA expression by human pancreatic stellate cells (PSCs) co-cultured with human PDAC cells. (**a**) Schematic representation of the indirect co-culture model: PSCs were seeded in the lower chambers and PDAC cells in the upper chambers of a transwell plate. (**b**) α-SMA-specific qRT-PCR analysis of mRNA derived from human PSCs co-cultured with Capan-1 (left panel) or HPAF-II (right panel) PDAC cells treated with MvDN30 and decoyMET^K842E^ in combination (Combo). Data are expressed as fold-change values relative to the untreated controls (NT). Each bar represents the average value ± SD. Student’s *t* test: *, *p* ≤ 0.05; **, *p* ≤ 0.01. (**c**) Schematic representation of the direct co-culture model: PSCs and PDAC cells were mixed in 1:3 proportion and plated in the same well. (**d**) Immunofluorescence analysis of α-SMA expression in human PSCs co-cultured with Capan-1 (left panel) or HPAF-II (right panel) cells. Cells were treated as above. Immunofluorescence staining was performed after 5 days of co-culture. Representative confocal sections show α-SMA in green and DAPI in blue. Scale bar is 50 µm. Data reported in figure are representative of at least three independent experiments.

To investigate the effect of MET pathway stimulation on tumor-stroma activation, we evaluated α-smooth-muscle actin (α-SMA) expression by human pancreatic stellate cells (PSCs) co-cultured with human pancreatic adenocarcinoma cells (Capan-1 or HPAF-II) and treated with the combination of MvDN30 and DecoyMET^K842E^ (Combo). After 5 days of co-culture with the indirect model (Figure 1a), the expression of α-SMA by PSCs was significantly reduced by the treatment, as assessed by quantitative real time (qRT-) PCR (Figure 1b). The effect of the Combo on α-SMA expression was also assessed in the co-culture direct model (Figure 1c), evaluating the amount of the protein by immunofluorescence staining (Figure 1d). The inhibitory effect of the HGF/MET axis on PSC activation was confirmed by analyzing the expression of fibroblast activation protein (FAP), which was reduced by the Combo treatment as shown in Figure S1. Since PSCs, like most fibroblasts, do not express MET [18], the inhibitory activity of the Combo treatment is an indirect effect exerted by tumor-secreted mediators able to transmit the signal from MET-expressing cancer cells to stromal cells.

### 3.2. Transcriptional Gene Expression Profiling of HPAF-II PDAC Cells in Response to HGF/MET Pathway Modulation

**Figure 2:**
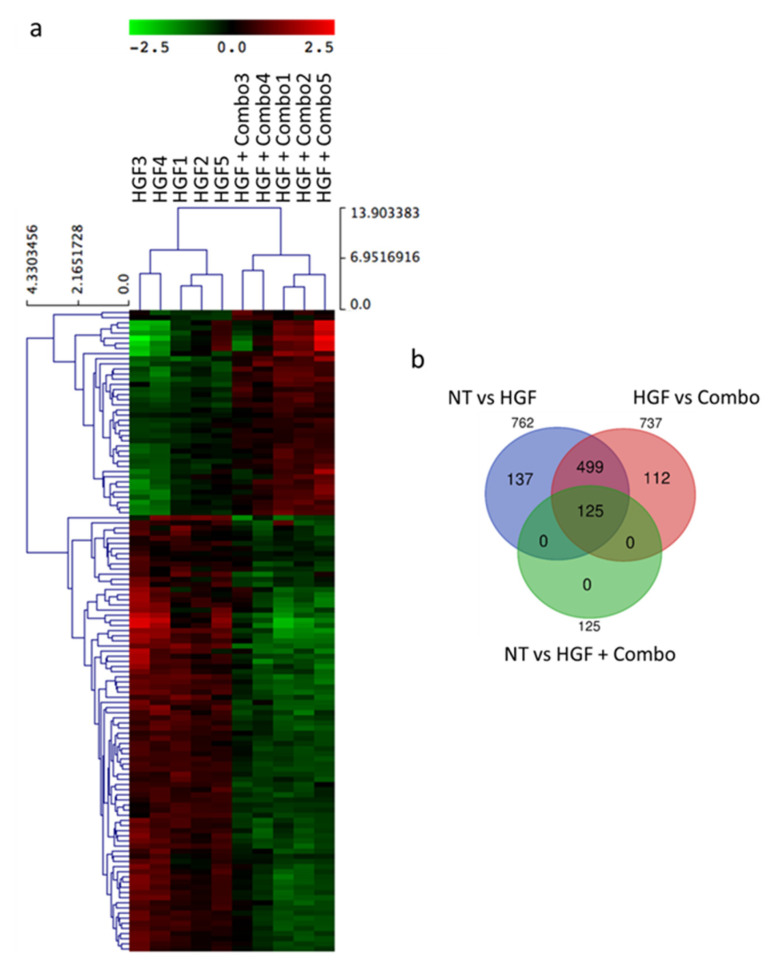
Gene expression analysis of HPAF-II cells in response to HGF/MET pathway modulation identifies 125 differentially expressed genes. (**a**) Hierarchical clustering of the 125 differential expressed genes obtained by comparing the expression profiles of HGF-stimulated HPAF-II cells (HGF 1–5) with those of HGF-stimulated HPAF-II cells treated with MvDN30 and DecoyMET^K842E^ in combination (HGF + Combo 1–5). (**b**) Venn diagram displaying overlap among sets of differentially expressed genes.

To understand how inhibition of the HGF/MET pathway in pancreatic cancer cells influences stroma activation, we performed a transcriptome analysis by RNA-sequencing-based gene expression analysis (RNAseq). The basal levels of gene expression in untreated HPAF-II cells were compared to the expression profiles of HPAF-II cells treated with: (i) HGF, (ii) Combo, or (iii) HGF and Combo. Total RNA was collected from five independent experiments, and differential gene expression analysis was performed. Transcriptional profiles of untreated versus HGF-treated cells identified 762 differentially expressed genes (Table S1), while untreated and Combo-treated cells originated superimposable profiles (Figure S2). By comparing the profiles of cells treated with HGF alone versus those treated with HGF + Combo, 125 differentially expressed genes were highlighted (Table S2 and Figure 2a). Notably, this subset of genes is totally included in the 762 genes modulated by HGF (Figure 2b).

### 3.3. TNC: A Candidate Mediator in HGF/MET-Dependent Tumor-Stroma Cross-Talk

**Figure 3:**
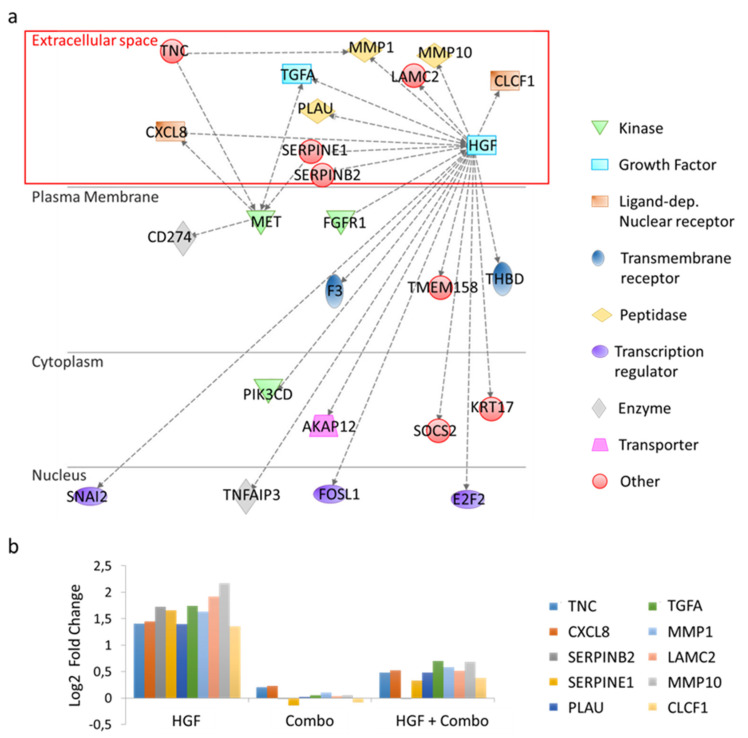
Ingenuity Pathway Analysis (IPA) of differentially regulated genes in HGF-treated versus HGF + Combo-treated HPAF-II cells. (**a**) Functional network generated by IPA including genes involved in indirect interactions with HGF and/or MET. Genes acting as intermediate bridges between HGF or MET are not shown. Nodes represent genes, dotted lines represent indirect biological relationships between nodes. The red box highlights genes encoding for proteins secreted in the extracellular space. (**b**) Bar graph reporting differential expression of genes encoding proteins secreted in the extracellular space. Values have been normalized versus untreated cells.

The above-mentioned subset of 125 genes was analyzed with Ingenuity Pathway Analysis (IPA) [27]. Notably, IPA gene set enrichment analysis by functional classes highlighted the involvement of these genes in cell movement, invasion, and migration (Table S3). Exploiting IPA’s mechanistic network tool, direct and indirect interactions among the selected genes were identified (Figure S3). By definition, ‘direct relationships’ occur when two molecules establish physical contacts with each other (i.e., by binding or phosphorylation), while ‘indirect interactions’ do not require a physical connection between the two molecules. We focused our attention on genes that were connected to MET or HGF by indirect interactions (Figure S4), without the intervention of intermediate bridging genes (Figure 3a). Moreover, since our goal was to identify soluble factors produced by cancer cells acting in a paracrine way on stromal cells, we only included differentially modulated genes encoding for proteins released in the extracellular space in the analysis. In this way, the number of candidates for the role of mediators in the tumor-stroma cross-talk was reduced to 10 genes (Figure 3b). The function of these genes is reported in Table S4. In this list, tenascin C (TNC) stands out for its pattern of expression and its role in the activation of the stromal compartment [28].

### 3.4. Validation of TNC Modulation Following Inhibition of the HGF/MET Axis in Pancreatic Cancer Cells In Vitro

**Figure 4:**
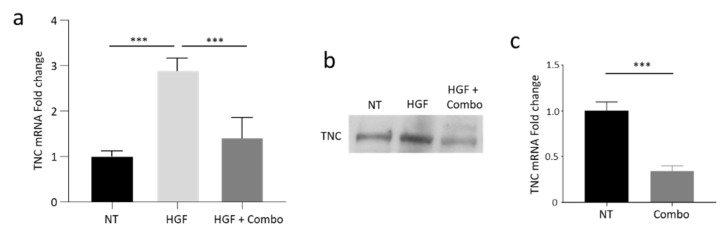
Activation of the HGF/MET axis modulates TNC expression in human PDAC cells in vitro. (**a**) TNC-specific qRT-PCR analysis of mRNA derived from HPAF-II human PDAC cells treated with HGF alone (HGF) or in combination with MvDN30 and DecoyMET^K842E^ (HGF + Combo). Data are expressed as fold-change values relative to the untreated controls (NT). Each bar represents the average value ± SD. ***, *p* ≤ 0.001. (**b**) Immunoblotting analysis of TNC expression in supernatants of HPAF-II cells treated as above. (**c**) TNC-specific qRT-PCR analysis of mRNA derived from Capan-1 human PDAC cells co-cultured with human PSCs and treated with the combination of MvDN30 and DecoyMET^K842E^ (Combo). Data are expressed as fold-change values relative to the untreated controls (NT). Each bar represents the average value ± SD. Student’s *t* test: ***, *p* ≤ 0.001. Data reported in figure are representative of at least three independent experiments.

To confirm the data generated by RNAseq, RNA was collected from HPAF-II cells treated as described above and analyzed by TNC-specific qRT-PCR. As shown in Figure 4a, TNC transcripts increased 2.9 fold after HGF stimulation and were down-modulated when MET activation was specifically inhibited by the Combo treatment. MvDN30 alone was also active, although with lower potency (Figure S5), in agreement with previous data showing a synergic effect of the Combo treatment compared to the antibody alone [19]. TNC modulation was confirmed at protein level, by immunoblotting performed on supernatants collected from HPAF-II cells treated as above (Figure 4b and Figure S6). Impairment of TNC expression induced by HGF/MET dual inhibition in PDAC cells was also observed in co-culture experiments (Figure 4c). Since PSCs do not express MET [18], HGF and/or Combo treatment were not active on the stroma compartment (Figure S7).

### 3.5. Validation of TNC Modulation and Reduction of Stroma Activation Following Inhibition of the HGF/MET Axis in PDAC Tumors In Vivo

**Figure 5:**
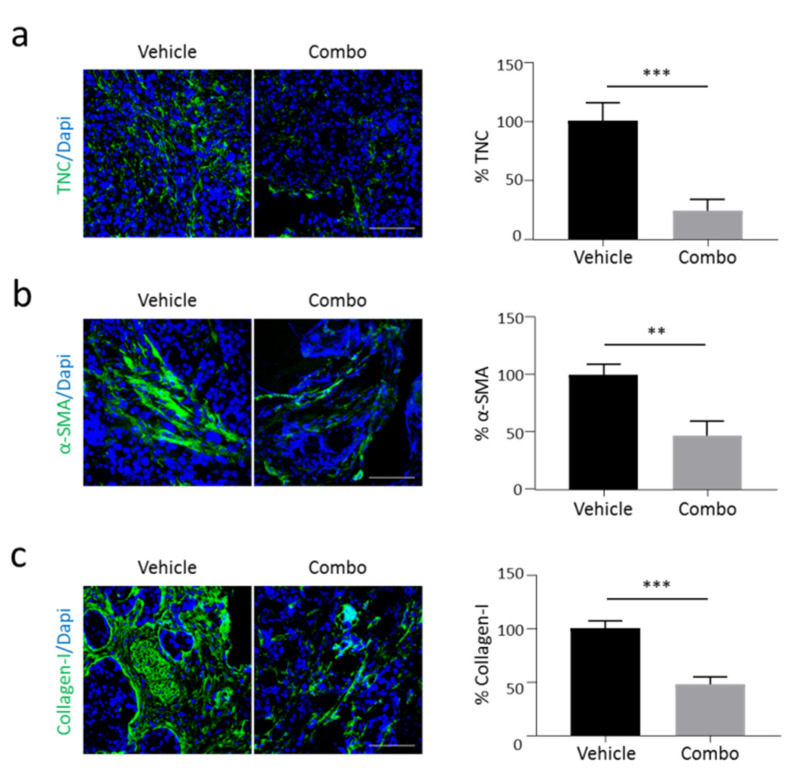
Activation of the HGF/MET axis modulates TNC expression and stroma activation in vivo. Primary tumors excised from hHGF-Ki mice injected orthotopically with Capan-1 human PDAC cells and treated with Vehicle or the MvDN30/DecoyMET^K842E^ combination (Combo) were analysed by immunofluorescence for: (**a**) TNC expression, (**b**) α-SMA expression, and (**c**) Collagen-I expression. Left panels: representative confocal sections showing TNC, α-SMA, and Collagen-I in green, DAPI in blue. Scale bars are 50 µm. Right panels: bar graphs showing the amount of TNC, α-SMA, or Collagen-I. Data are expressed as percentage of the control. Student’s *t* test: **, *p* ≤ 0.01; ***, *p* ≤ 0.001. Data reported in figure are representative of at least two independent experiments.

To evaluate the effect of the HGF/MET inhibition on stroma rewiring in vivo, luciferase-expressing Capan-1 cells were orthotopically injected into the pancreas of hHGF-Ki immunocompromised mice. This transgenic model allows the reconstitution of a fully active HGF/MET axis [29], bypassing the limitations due to poor interaction between ligand/receptor pairs of different species [30,31]. Cell engraftment was evaluated by total body luminescence analysis and mice were randomly assigned to two treatment groups: Vehicle and Combo. Five weeks after cell injection, mice were sacrificed, and the excised tumors underwent immunofluorescence analysis. In line with our in vitro data, we observed a reduction in TNC expression in tumors treated with HGF/MET inhibitors as compared to Vehicle (Figure 5a). This decrease in TNC expression matched with a dramatic reduction in α-SMA-positive cells in the stroma compartment (Figure 5b); concomitantly, as a consequence of the diminished PSCs activation, the deposition of Collagen-I in the extracellular environment was significantly reduced (Figure 5c). The effect of Combo treatment on stroma activation was superior compared to the activity exerted by MvDN30 alone (Figure S8).

### 3.6. HGF/MET-Dependent Tumor-Stroma Cross-Talk Is Mediated by TNC

**Figure 6:**
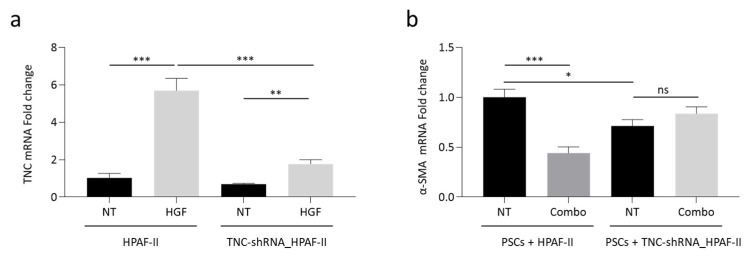
Knocking-down of TNC expression blunts MET-mediated stromal activation. (**a**) TNC-specific qRT-PCR analysis of mRNA derived from HPAF-II cells (wild-type or genetically engineered to express TNC-shRNAs) treated with HGF. Data are expressed as fold-change values relative to the untreated control (wild-type HPAF-II). (**b**) α-SMA-specific qRT-PCR analysis of mRNA derived from human PSCs co-cultured with HPAF-II wild-type or genetically engineered to express TNC-shRNAs, treated with MvDN30 and decoyMET^K842E^ in combination (Combo). Data are expressed as fold-change values relative to untreated control (PSCs co-cultured with wild-type HPAF-II). NT, untreated controls. Each bar represents the average value ± SD. Student’s *t* test: *, *p* ≤ 0.05; **, *p* ≤ 0.01; ***, *p* ≤ 0.001; ns, not significant. Data reported in figure are representative of at least two independent experiments.

The role played by TNC in MET-mediated tumor-stroma cross-talk has been further investigated by knocking-down TNC expression. Specific TNC-targeting shRNAs were delivered to HPAF-II cells by lentiviral vector technology. In genetically modified HPAF-II cells, TNC-shRNAs strongly counteracted the up-modulation of TNC expression triggered by HGF (Figure 6a). TNC-shRNA_HPAF-II cells were challenged in the indirect co-culture model with PSCs. Data reported in Figure 6b show that α-SMA expression was reduced in PSCs cells co-cultured with genetically modified HPAF-II cells. Moreover, α-SMA expression was not further reduced by the Combo treatment. These results point at TNC as a major molecular mediator of the stromal rewiring orchestrated by MET activation.

## 4. Discussion

In this study, we investigated the effect of HGF/MET axis modulation on pancreatic-cancer-cell signature, focusing on the differential expression of TNC.

TNC is an extracellular hexameric glycoprotein interacting with extracellular matrix components such as fibronectin, syndecan, membrane proteoglycans, and cell surface receptors involved in cell adhesion [32]. Its expression occurs in a tightly regulated manner during embryogenesis [33,34]. In the adult, TNC expression is restricted to the stem cell niche and the sites of epithelial–mesenchymal interaction [35]. Interestingly, TNC expression increases during tissue regeneration processes occurring after injury and during inflammation [36]. Functional correlation of TNC expression in the process of angiogenesis has been highlighted, both in physiological and in pathological conditions [37]. It has been reported that TNC supports proliferation and inhibits differentiation of cancer stem cells [38]. Moreover, TNC influences cytoskeleton remodeling, interfering with actin stress fiber polymerization and affecting expression and/or localization of actin binding proteins [39]. A large body of evidence highlights the involvement of TNC in metastasis formation, in particular favoring epithelial to mesenchymal transition of transformed cells [39]. In support of cancer cell dissemination, TNC displays pro-migratory effects and promotes cell invasion, as it is expressed, depending on the malignant status of the tumor, at the invasive front of the tumor lesions. In PDAC, the abundant fibrotic tissue around tumor cells is rich in TNC, and its expression increases during disease progression from a low-grade precursor lesion to the invasive cancer [40].

MET has been identified as a master gene regulating the ‘invasive growth’ program [16]. This is a complex, integrated, and finely tuned network of cellular signaling leading to proliferation, motility, invasion, and survival. The essential role of MET signaling during embryonic development has been accurately described [41], as well as the relationship between stem status and MET expression [42]. These physiological activities are exploited during cell transformation. MET activation can play the role of driver of the malignancy and/or it can take on fundamental functions related to the ability of cancer cells to overcome stress conditions [43]. These activities take place in the primary lesion and during the metastatic process. MET expression/activation strongly correlates with the malignant grade of the tumor, as well as with the prognosis of the disease [44]. It is thus impressive how much the expression/functional pattern of TNC is superimposable to that of MET. Our data indicate that HGF secreted by the activated stroma induces MET activation in tumor cells, and these respond by increasing TNC secretion. TNC positively influences the activation of the surrounding stroma, further sustaining the transformed phenotype and exacerbating the disease. We thus suggest that a positive feed-back loop may be established between the tumor and the stroma, pointing at TNC as a major player in the cross-talk. Moreover, a signal hierarchy can be defined, where MET is upstream of TNC; the latter could then represent the operative arm through which MET orchestrates the invasive growth response.

As a factor secreted by the tumor microenvironment, TNC has been largely involved in ‘non-cell-autonomous functions’ related to both initiation of tumorigenesis and progression to metastasis. While it is clear that stroma-secreted TNC acts as a pro-invasive factor on pancreatic cancer cells, little is known about the role that this secreted factor could exert on the microenvironment, and in particular on PSCs. Our in vitro co-culture system, although simplified compared to a real tumor, provided relevant information about the effects of cancer cells on the stroma. By expressing HGF, PSCs act in a paracrine way on the tumor cells expressing MET. MET activation induces TNC secretion by tumor cells and this may influence the function of the surrounding stroma, including modulation of PSCs activation. As PSCs do not express the MET receptor, inhibition of the HGF/MET axis by the Combo treatment cannot directly influence the PSC status, but must be the consequence of a cross-talk between the tumor and the stroma compartment, which probably occurs through TNC.

In contrast to the large number of studies reporting TNC expression by the tumor microenvironment, TNC production by cancer cells has been poorly investigated. In a previous study conducted in a breast cancer model, tumor-derived TNC was reported to be a relevant component of the metastatic niche, playing a distinct role in supporting the fitness of disseminating cancer cells [45]. This function is in line with our data showing that tumors treated with inhibitors of the HGF/MET axis—which express low level ofTNC—are strongly impaired in their metastatic potential [19,20].

Blocking of the HGF/MET axis by the Combo treatment was extremely specific, with untreated and Combo-treated cancer cells giving rise to a superimposable expression profile. Therefore, the observed modulation of PSCs activation must be ascribed to MET-activated functions. Moreover, when comparing the two HGF-stimulated groups (Combo-treated or not), the differentially modulated genes are all included in the larger group defined by the HGF-responsive genes, indicating that solely these genes mediate the phenotype reversion induced by the Combo treatment in HGF-stimulated cells. In this frame, TNC represents a possible mediator of the response, being a secreted protein that is down-modulated when MET activation is switched-off. The molecular mechanism underling this regulation must rely on the control of TNC mRNA levels. It has been reported that ETS is among the transcription factors that orchestrate TNC promoter activity [46]. Interestingly, ETS-1 is in the list of genes up-regulated by HGF, so we can speculate that the modulation of TNC expression occurs through the control of the activity of this transcription factor.

## 5. Conclusions

In conclusion, this work highlights a new role for TNC, representing a newly discovered link between MET-expressing cancer cells and the stroma compartment.

## Data Availability

RNAseq raw data are available on request from the corresponding author.

## Supplementary Materials

The following are available online at https://www.mdpi.com/article/10.3390/cancers13143519/s1, Figure S1: FAP-specific real-time PCR analysis of mRNA derived from human PSCs co-cultured with title PDAC cells, Figure S2: Principal component analysis (PCA) of the normalized RNAseq data, Figure S3: Functional network generated by ingenuity pathway analysis (IPA) of differentially regulated genes in HGF-treated versus HGF + Combo-treated HPAF-II cells, including direct and indirect interactions, Figure S4: Functional network generated by ingenuity pathway analysis (IPA) of differentially regulated genes in HGF-treated versus HGF + Combo-treated HPAF-II cells, including only indirect interactions, Figure S5: TNC-specific real-time PCR analysis of mRNA derived from human HPAF-II treated with MvDN30 or Combo, Figure S6: Analysis of TNC secretion by human PDAC cells treated with HGF or HGF + Combo, Figure S7: TNC-specific real-time PCR analysis of mRNA derived from human PSCs treated with HGF and/or Combo, Figure S8: Analysis of stromal activation in PDAC tumors treated with MvDN30 or Combo, Table S1: List of differentially expressed genes (untreated vs. HGF-treated HPAF-II cells), Table S2: List of differentially expressed genes (untreated vs. HGF + Combo-treated HPAF-II cells), Table S3: IPA analysis of differentially expressed genes (untreated vs. HGF + Combo-treated HPAF-II cells), Table S4: Candidate genes mediating tumor-stroma cross-talk.

## Abbreviations

α-SMA	Alpha-smooth muscle actin
FAP	Fibroblast-activating protein
HGF	Hepatocyte growth factor
IPA	Ingenuity pathway analysis
MFI	Mean fluorescence intensity
PDAC	Pancreatic ductal adenocarcinoma
PSCs	Pancreatic stellate cells
qRT-PCR	Quantitative real-time polymerase chain reaction
RNAseq	RNA-sequencing-based gene expression analysis
SCID	Severe combined immunodeficiency
SD	Standard deviation
SEM	Standard error of the mean
TNC	Tenascin C
