# Supplementary material for: HGF/MET Axis Induces Tumor Secretion of Tenascin-C and Promotes Stromal Rewiring in Pancreatic Cancer
Source: Cancers (Basel). 2021 Jul 14;13(14):3519. doi: 10.3390/cancers13143519 (PMC8305254; doi:10.3390/cancers13143519)
Supplement: Supplementary file 1 [file cancers-13-03519-s001.zip › cancers-1224808-suppl. figures 1-8.pdf]

Supplementary Materials: HGF/MET Axis Induces Tumor Secretion of Tenascin-C and Promotes Stromal Rewiring in Pancreatic Cancer

Chiara Modica, Martina Olivero, Francesca Zuppini, Melissa Milan, Cristina Basilico and Elisa Vigna

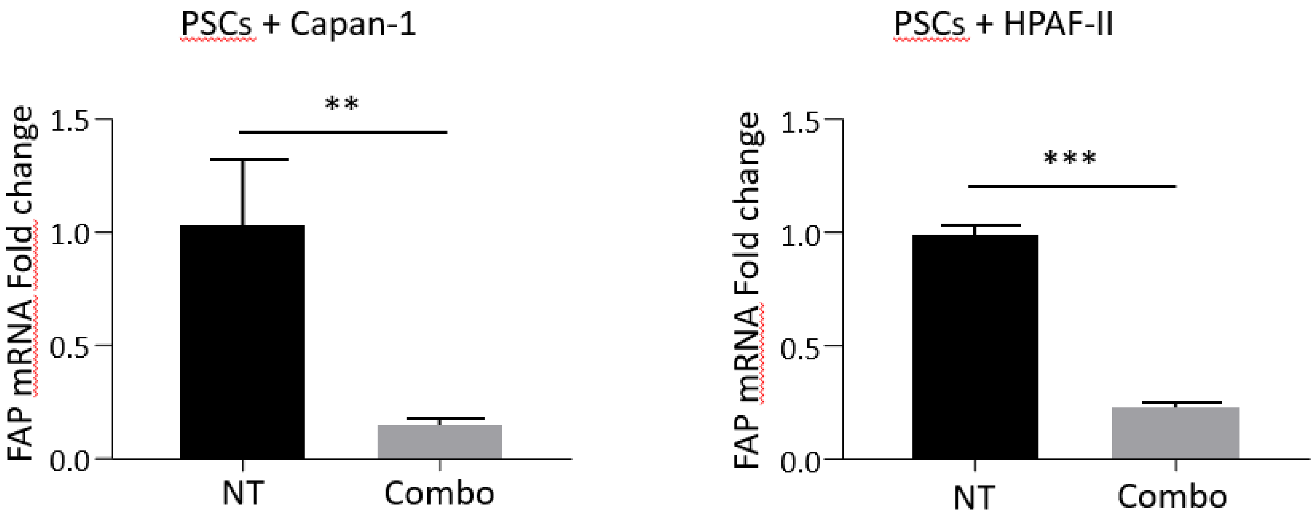

Figure S1. FAP-specific qRT-PCR analysis of mRNA derived from human PSCs co-cultured with Capan-1 (left panel) or HPAF-II (right panel) PDAC cells and treated with MvDN30 and DecoyMET^{K842E} in combination (Combo). Data are expressed as fold change values relative to the untreated controls (NT). Each bar represents the average value ± SD. Student T test: **, $p \leq 0.01$; ***, $p \leq 0.001$. Data reported in figure are representative of at least 3 independent experiments.

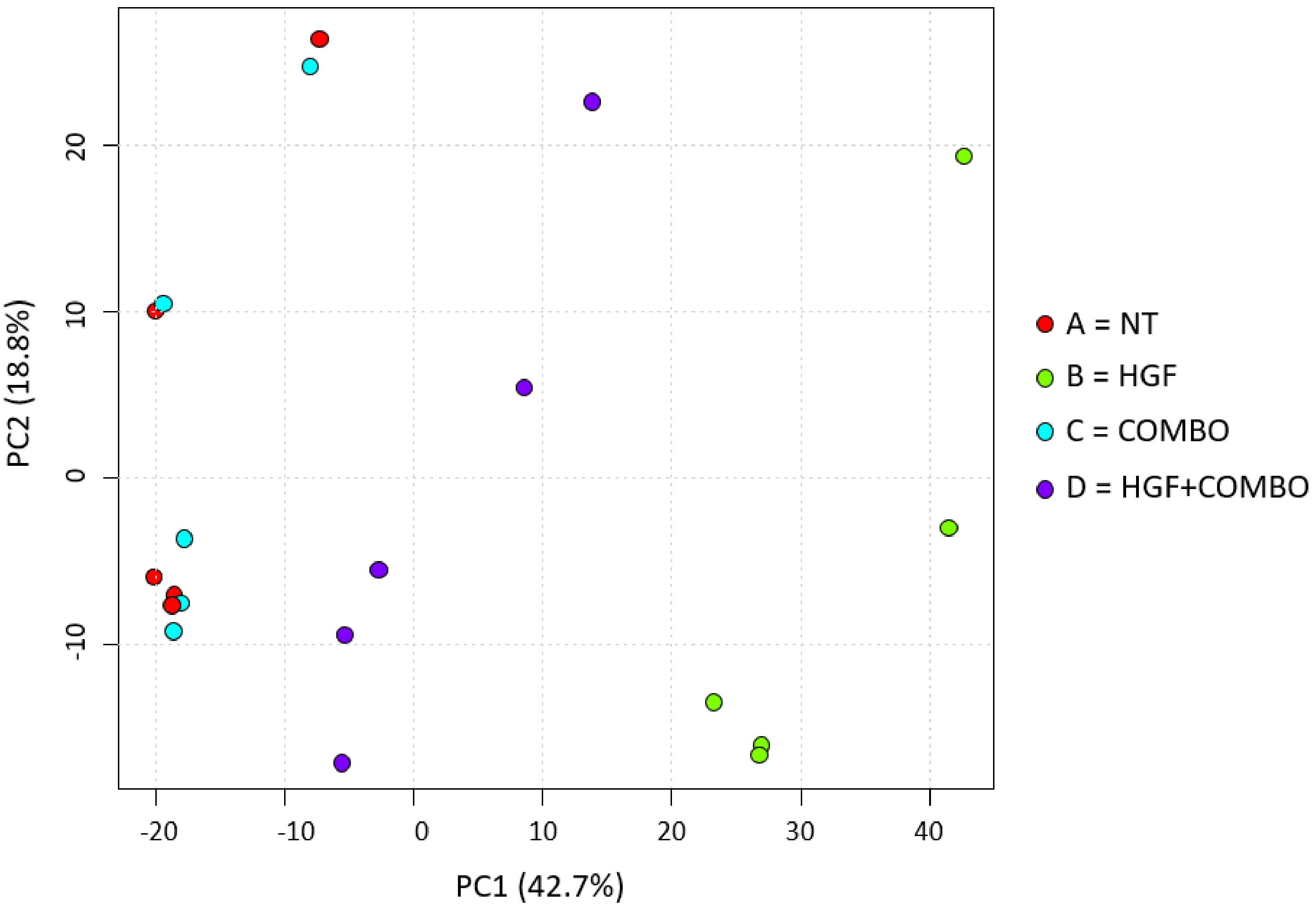

Figure S2. Principal Component Analysis (PCA) of the normalized RNAseq data. The plot shows the variance of 5 replicates for each experimental treatment. Samples are color-coded by the treatment. Each dot represents a sample.

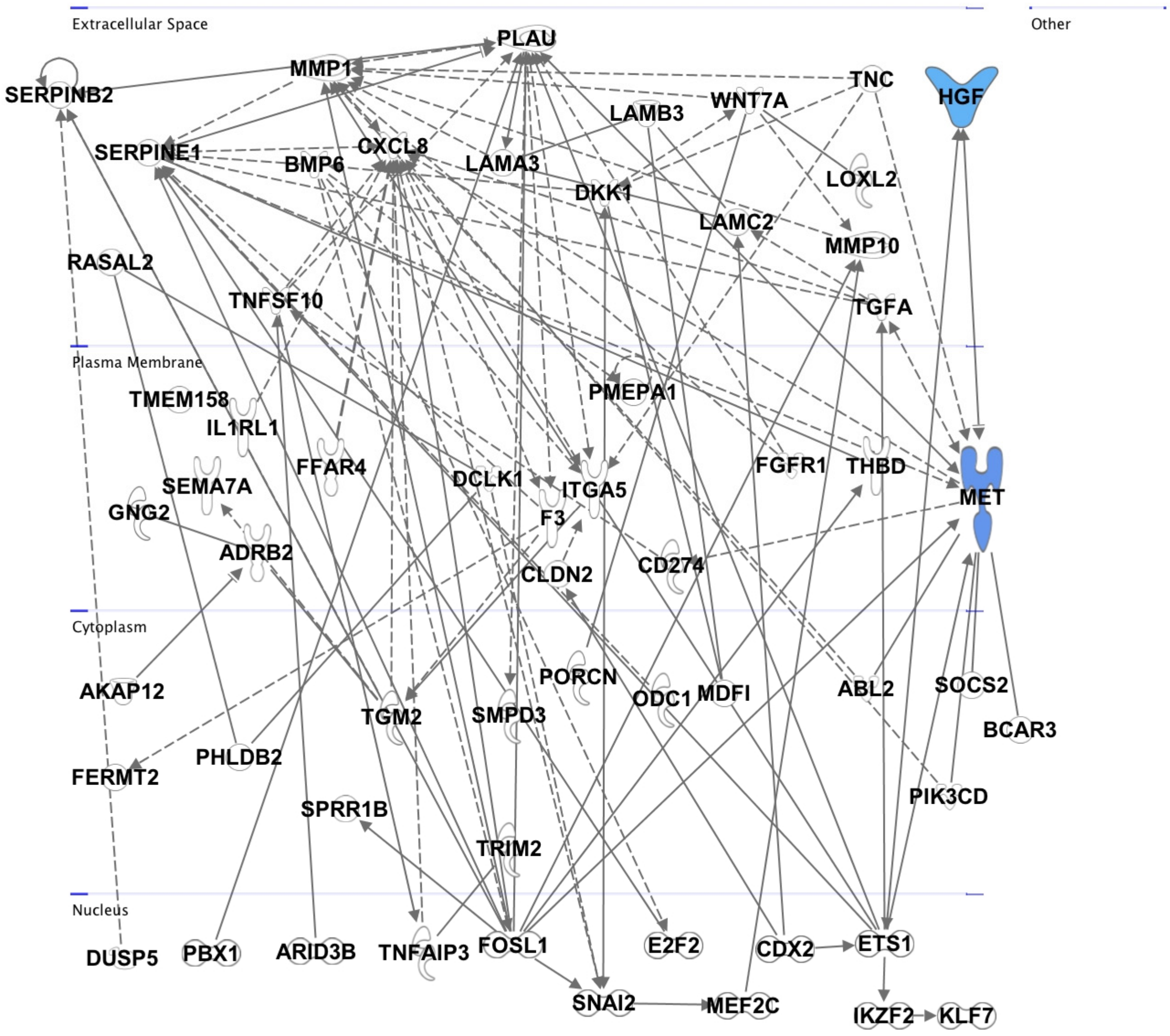

Figure S3. Functional network generated by Ingenuity Pathway Analysis (IPA) of differentially regulated genes in HGF-treated versus HGF + Combo-treated HPAF-II cells. The network includes genes involved in direct or indirect interactions with HGF and/or MET. Nodes represent genes; solid and dotted lines represent direct and indirect biological relationships between nodes, respectively.

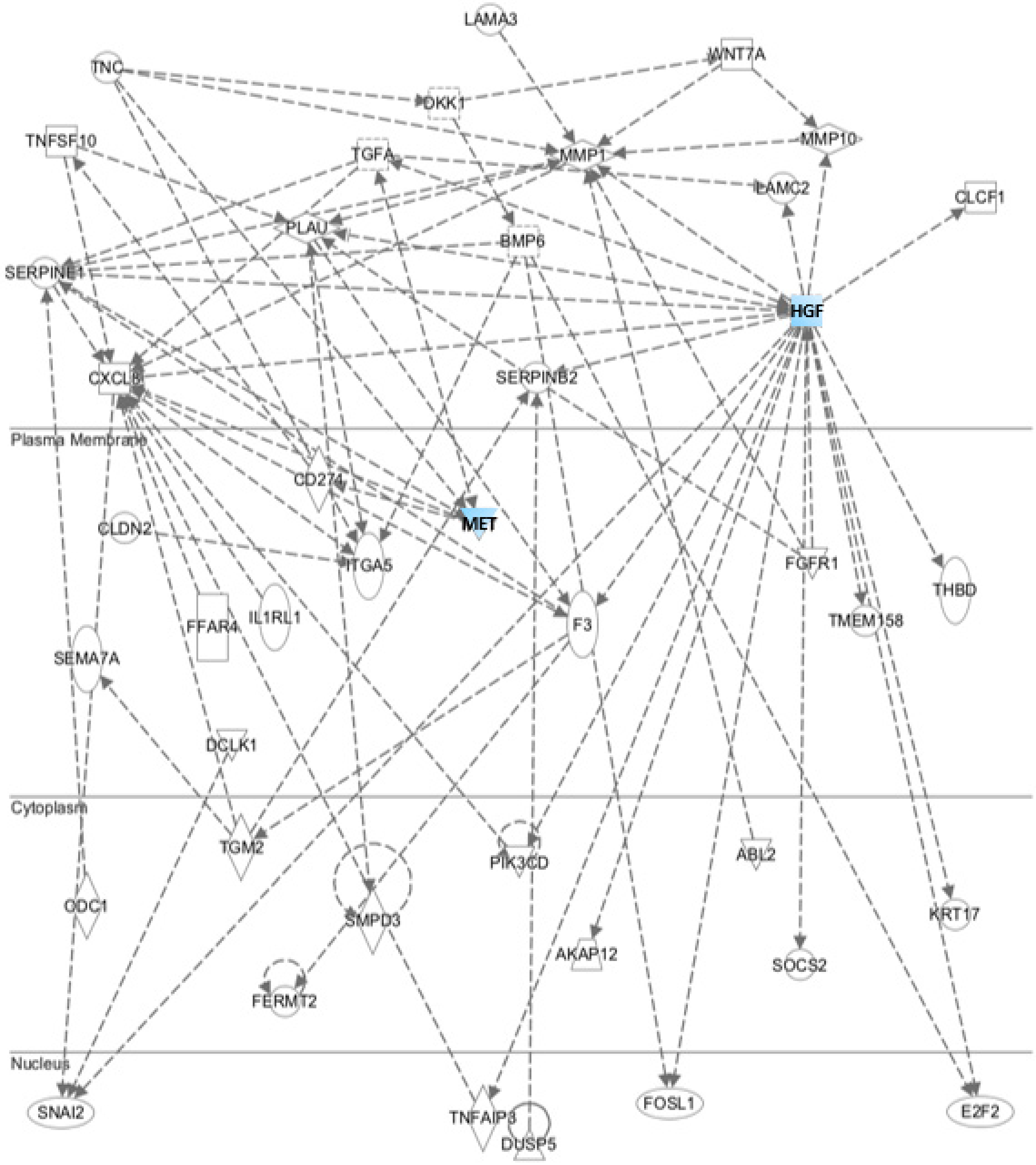

Figure S4. Functional network generated by Ingenuity Pathway Analysis (IPA) of differentially regulated genes in HGF-treated versus HGF + Combo-treated HPAF-II cells. The network includes genes involved in indirect interactions with HGF and/or MET. Nodes represent genes; dotted lines represent indirect biological relationships between nodes. Genes acting as intermediate bridges between HGF/MET and TNC are included in the network.

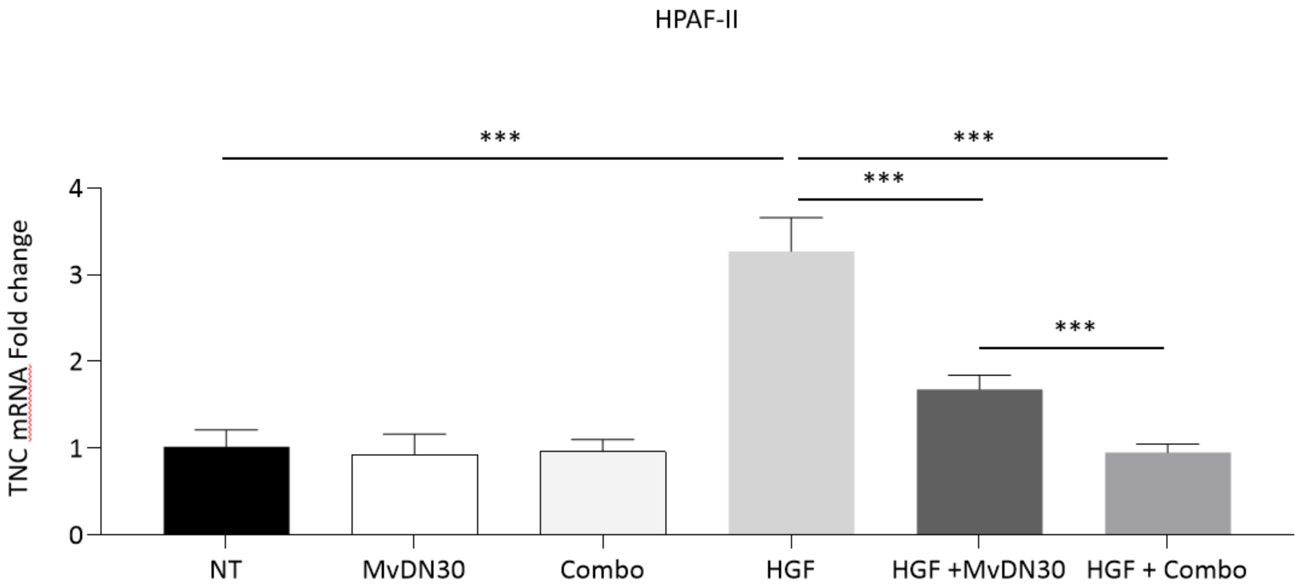

Figure S5. TNC-specific qRT-PCR analysis of mRNA derived from human HPAF-II treated with MvDN30 alone or MvDN30 and DecoyMET^{K842E} in combination (Combo), with or without HGF. Data are expressed as fold change values relative to the untreated controls (NT). Each bar represents the average value ± SD. Student T test: ***, p ≤ 0.001. Data reported in figure are representative of at least 3 independent experiments.

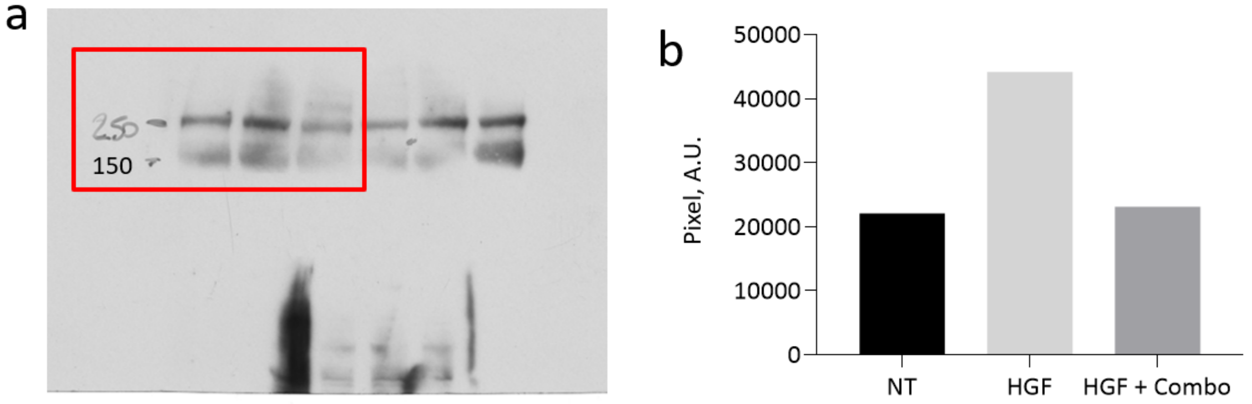

Figure S6. Activation of the HGF/MET axis modulates TNC secretion in human PDAC cells in vitro. Immunoblotting analysis of TNC expression in the supernatants of HPAF-II cells treated with HGF alone (HGF) or in combination with MvDN30 and DecoyMET^{K842E} (HGF + Combo). NT: Not treated cells. a) Raw data. b) Graph reporting TNC bands intensity measured by densitometry.

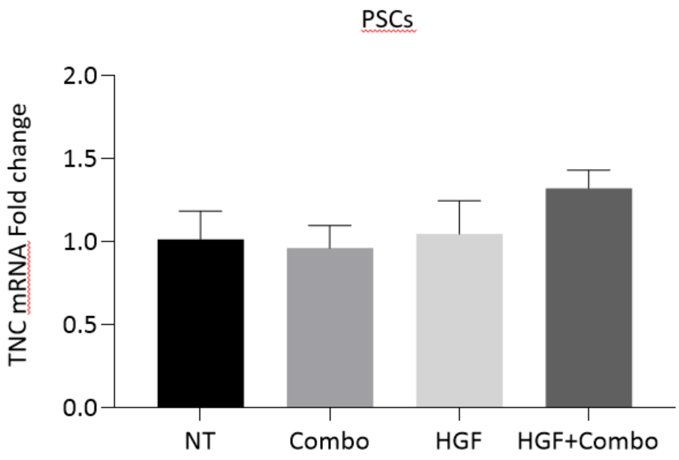

Figure S7. TNC-specific qRT-PCR analysis of mRNA derived from human PSCs treated with MvDN30 and DecoyMET^{K842E} in combination (Combo), HGF, or HGF+Combo. Data are expressed as fold change values relative to the untreated controls (NT). Each bar represents the average value ± SD. No statistically relevant differences were scored (Student T test). Data reported in figure are representative of at least 2 independent experiments.

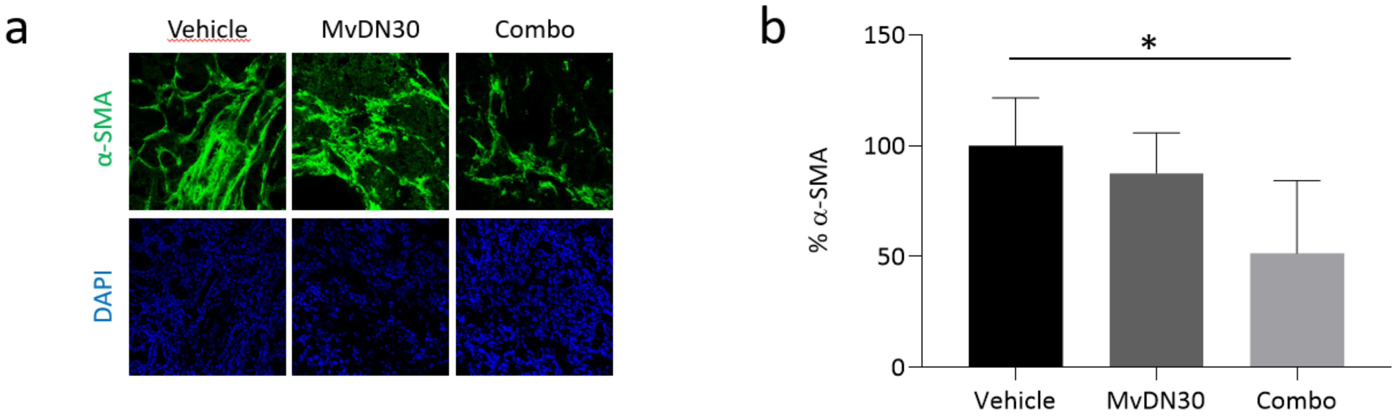

Figure S8. MvDN30 and decoyMET^{K842E} in combination attenuate stromal activation *in vivo* more effectively than MvDN30 alone. Primary tumors excised from hHGF-Ki mice injected orthotopically with HPAF-II human PDAC cells and treated with Vehicle, MvDN30, or MvDN30 and DecoyMET^{K842E} in combination (Combo) were analysed for α-SMA expression by immunofluorescence. a) Representative confocal sections showing α-SMA in green and DAPI in blue. b) Bar graphs showing the quantification of α-SMA signal. Data are expressed as percentage of the control ± SEM. Student T test: *, $p \leq 0.05$. Data reported in figure are representative of at least 2 independent experiments.
