# Supplementary material for: HGF/MET Axis Induces Tumor Secretion of Tenascin-C and Promotes Stromal Rewiring in Pancreatic Cancer
Source: Cancers (Basel). 2021 Jul 14;13(14):3519. doi: 10.3390/cancers13143519 (PMC8305254; doi:10.3390/cancers13143519)
Supplement: Supplementary file 1 [file cancers-13-03519-s001.zip › Table S1_Modica et al..pdf]

Suppl. Table S1: differentially expressed genes (untreated vs HGF-tr

symbol	ensembl gene ID	baseMean
DCLK1	ENSG00000133083	80.09512513
SEMA7A	ENSG00000138623	660.8415079
KRT6A	ENSG00000205420	258.453552
SERPINB2	ENSG00000197632	38.70119509
IL1RL1	ENSG00000115602	121.5263427
NAV3	ENSG00000067798	137.5987388
FOSL1	ENSG00000175592	3550.604451
G0S2	ENSG00000123689	404.0778416
SPRR1B	ENSG00000169469	78.46740221
SNAI2	ENSG00000019549	677.5414399
MDGA1	ENSG00000112139	174.3753749
AKAP12	ENSG00000131016	9348.867452
MMP10	ENSG00000166670	465.9168215
ADRB2	ENSG00000169252	175.5422005
CGB5	ENSG00000189052	283.0807811
ETS1	ENSG00000134954	4046.295936
GFOD1	ENSG00000145990	401.3280088
ZBED2	ENSG00000177494	1490.207275
LOXL2	ENSG00000134013	204.5301296
WNT7A	ENSG00000154764	84.15711755
RFX8	ENSG00000196460	28.53968987
FERMT2	ENSG00000073712	169.7284614
C12orf56	ENSG00000185306	40.60165631
KRTAP3-1	ENSG00000212901	46.58484262
SH2D5	ENSG00000189410	140.8666619
KIAA1462	ENSG00000165757	116.7288908
PTRF	ENSG00000177469	550.3948887
TNFAIP3	ENSG00000118503	428.39922
SERPINE1	ENSG00000106366	301.1912867
ARID3B	ENSG00000179361	336.8937291
SPOCD1	ENSG00000134668	200.5265721
LAMC2	ENSG00000058085	18541.09977
KIAA1549L	ENSG00000110427	489.8754674
FST	ENSG00000134363	14.07590594
CHST11	ENSG00000171310	1120.380823
KLF7	ENSG00000118263	676.2839586
PEAR1	ENSG00000187800	501.4462066
FAM83A	ENSG00000147689	2896.599308
CD274	ENSG00000120217	199.3455825
TMEM158	ENSG00000249992	84.43891325
OSBP2	ENSG00000184792	455.7582992

BCAR3	ENSG00000137936	1370.100618
GPAT3	ENSG00000138678	3075.01993
THBD	ENSG00000178726	2775.713084
GLIPR1	ENSG00000139278	625.8757951
CGB8	ENSG00000213030	60.39984965
PHLDB2	ENSG00000144824	276.9070995
RHOB	ENSG00000143878	2332.300595
NRIP3	ENSG00000175352	142.7660463
RELN	ENSG00000189056	122.5406239
MARCH_4	ENSG00000144583	22.64322463
PIK3CD	ENSG00000171608	28.73758359
DKK1	ENSG00000107984	12664.45256
LRRC8C	ENSG00000171488	77.64135067
LRAT	ENSG00000121207	247.0259409
TGFA	ENSG00000163235	5058.000843
ARHGAP29	ENSG00000137962	3607.270519
UPP1	ENSG00000183696	1367.601191
TM4SF19	ENSG00000145107	137.1040702
RASD1	ENSG00000108551	19.87844779
TNC	ENSG00000041982	503.6030154
S100A2	ENSG00000196754	153.1717377
AREG	ENSG00000109321	3002.996243
CPA4	ENSG00000128510	223.7113163
SPRR3	ENSG00000163209	54.59821743
STK17A	ENSG00000164543	1596.855953
NAV2	ENSG00000166833	213.4289468
ARL4C	ENSG00000188042	1004.111456
VEGFC	ENSG00000150630	34.75797267
KRT17	ENSG00000128422	21.71244596
ANTXR2	ENSG00000163297	2243.476649
TNS4	ENSG00000131746	3708.595376
BMP6	ENSG00000153162	39.87472174
MMP1	ENSG00000196611	8713.390914
CORO2B	ENSG00000103647	78.3355581
ITGA2	ENSG00000164171	11158.52834
TBC1D2	ENSG00000095383	792.8349429
TMCC3	ENSG00000057704	762.8298163
F3	ENSG00000117525	27344.96982
RP1	ENSG00000104237	32.13207889
HBEGF	ENSG00000113070	969.0558033
DHRS9	ENSG00000073737	15045.50871
E2F7	ENSG00000165891	929.4467623
IFFO2	ENSG00000169991	1231.809673
AKAP2	ENSG00000241978	3958.887352

ITGA5	ENSG00000161638	207.4086583
MDFI	ENSG00000112559	705.6600552
GJB3	ENSG00000188910	2580.424394
LAMB3	ENSG00000196878	8318.339328
CAPRIN2	ENSG00000110888	5237.401161
TUBB6	ENSG00000176014	1678.107073
ITPRIP	ENSG00000148841	562.806456
HMGA2	ENSG00000149948	3812.659512
FGFR1	ENSG00000077782	853.3531286
IL11	ENSG00000095752	24.14517382
PLCXD2	ENSG00000240891	288.2203478
ERRFI1	ENSG00000116285	15337.11633
FOXD1	ENSG00000251493	356.3643771
PHACTR3	ENSG00000087495	729.3201835
CYTH4	ENSG00000100055	37.44419162
EREG	ENSG00000124882	3612.583599
EVA1A	ENSG00000115363	95.89933719
GALNT9	ENSG00000182870	111.9427417
CXCL8	ENSG00000169429	257.3687132
CREB5	ENSG00000146592	133.2013013
LETM2	ENSG00000165046	758.3268383
PMEPA1	ENSG00000124225	3097.239154
SOCS2	ENSG00000120833	153.2967765
CD59	ENSG00000085063	9289.458711
IL7R	ENSG00000168685	140.1606268
SLC9A2	ENSG00000115616	257.6700061
SOCS3	ENSG00000184557	457.4106279
CLCF1	ENSG00000175505	578.1229534
HS3ST3A1	ENSG00000153976	21.61119179
FRMD6	ENSG00000139926	595.7275783
CCDC71L	ENSG00000253276	1181.563717
DUSP5	ENSG00000138166	2927.227311
EDN1	ENSG00000078401	169.5985328
FGFBP1	ENSG00000137440	962.035448
FHL2	ENSG00000115641	6691.064738
SDC4	ENSG00000124145	8964.379106
RASAL2	ENSG00000075391	1146.685202
DUSP7	ENSG00000164086	1256.787312
NOCT	ENSG00000151014	140.090613
SLC2A1	ENSG00000117394	10677.96222
SLC4A7	ENSG00000033867	1429.76982
DUSP4	ENSG00000120875	1649.137925
ARNTL2	ENSG00000029153	5462.86694
PLAU	ENSG00000122861	6310.143716

RTL1	ENSG00000254656	7.851088271
EPHA2	ENSG00000142627	7741.151803
GJB4	ENSG00000189433	459.7381538
ZNF697	ENSG00000143067	619.2257757
PMAIP1	ENSG00000141682	1135.021358
JAG1	ENSG00000101384	6275.339758
ODC1	ENSG00000115758	7273.286623
PHLDA2	ENSG00000181649	2124.720434
SOCS1	ENSG00000185338	18.17782565
PORCN	ENSG00000102312	807.0421602
ABL2	ENSG00000143322	441.8146285
AXL	ENSG00000167601	856.814236
IER3	ENSG00000137331	3634.344904
GRB10	ENSG00000106070	1595.979352
PAQR5	ENSG00000137819	930.4050492
ALOXE3	ENSG00000179148	104.2438862
SLC35F3	ENSG00000183780	16.73303701
WFDC3	ENSG00000124116	91.16983131
KIRREL	ENSG00000183853	3035.229295
ADAM19	ENSG00000135074	4063.048529
PRDM1	ENSG00000057657	195.8879781
PHLDA1	ENSG00000139289	15131.17237
MBOAT2	ENSG00000143797	1894.929248
ELL2	ENSG00000118985	1461.66982
ARSI	ENSG00000183876	21.49706761
TMC7	ENSG00000170537	1037.223057
DLC1	ENSG00000164741	867.0103112
TNFRSF10A	ENSG00000104689	533.5409884
FLNA	ENSG00000196924	34417.39455
SERPINB9	ENSG00000170542	807.8994739
S100A16	ENSG00000188643	6864.548404
KRT6B	ENSG00000185479	7.105228114
NT5E	ENSG00000135318	3558.108369
MCL1	ENSG00000143384	7276.588447
FSTL3	ENSG00000070404	1299.139823
EGR1	ENSG00000120738	2806.532747
C3orf67	ENSG00000163689	274.1214162
MCC	ENSG00000171444	999.2177142
CTGF	ENSG00000118523	749.5499904
DENND2C	ENSG00000175984	26.3556904
SH2B3	ENSG00000111252	634.2789241
C8orf4	ENSG00000176907	33.96716498
PLEC	ENSG00000178209	23892.03106
CGB7	ENSG00000196337	86.36653447

LAMA3	ENSG00000053747	6895.331961
PEA15	ENSG00000162734	1887.570907
LDLR	ENSG00000130164	5890.184265
STC1	ENSG00000159167	34.28671693
ZNF185	ENSG00000147394	2121.919882
USP53	ENSG00000145390	1894.111905
C16orf52	ENSG00000185716	824.934301
PDGFB	ENSG00000100311	255.5578185
STRIP2	ENSG00000128578	638.8919226
BTBD19	ENSG00000222009	59.33663038
CYR61	ENSG00000142871	341.9175762
ZEB1	ENSG00000148516	44.86957967
KCNF1	ENSG00000162975	23.75277968
CAV1	ENSG00000105974	834.1359264
HRH1	ENSG00000196639	1450.506495
CAV2	ENSG00000105971	1102.222368
BHLHA15	ENSG00000180535	44.09415436
SLC25A32	ENSG00000164933	1002.904643
MICAL2	ENSG00000133816	3164.984146
NOG	ENSG00000183691	9.671633758
PTHLH	ENSG00000087494	84.86964089
LCP1	ENSG00000136167	9.166054923
MAGEB17	ENSG00000182798	91.07192844
PVR	ENSG00000073008	3694.455646
THBS1	ENSG00000137801	1378.714728
GPSM1	ENSG00000160360	221.314844
MFSD2A	ENSG00000168389	119.9668075
PPP1R15A	ENSG00000087074	1350.757077
NAGS	ENSG00000161653	336.111964
RSC1A1	ENSG00000215695	74.25648995
DUSP1	ENSG00000120129	472.7731644
DNAH11	ENSG00000105877	245.3293135
LONRF3	ENSG00000175556	443.7598555
GJB2	ENSG00000165474	1515.26844
GPRC5A	ENSG00000013588	26032.74086
PRSS22	ENSG00000005001	3161.597577
MPO	ENSG00000005381	6.669619999
DNTTIP1	ENSG00000101457	4933.092308
ADAMTSL4	ENSG00000143382	34.57168517
PLK3	ENSG00000173846	382.2972834
PRDM8	ENSG00000152784	331.0923332
IGFN1	ENSG00000163395	14.27786496
SPTBN5	ENSG00000137877	34.47057622
B4GALT6	ENSG00000118276	797.1896344

MAP1B	ENSG00000131711	57.1632979
EZR	ENSG00000092820	16206.97279
CDC42EP3	ENSG00000163171	848.2133585
ZYX	ENSG00000159840	4097.848506
RAI14	ENSG00000039560	1058.100269
AEN	ENSG00000181026	1508.824281
CEP170	ENSG00000143702	688.9868095
APBB2	ENSG00000163697	1127.716552
WNT7B	ENSG00000188064	3273.101958
HMGCS1	ENSG00000112972	3531.92389
ACTB	ENSG00000075624	133560.2629
IL33	ENSG00000137033	15.0413393
DUSP6	ENSG00000139318	8552.176636
WNT9A	ENSG00000143816	25.8330322
EMP1	ENSG00000134531	4125.774091
PAPPA	ENSG00000182752	449.5093081
HAS2	ENSG00000170961	347.4276953
PDLIM2	ENSG00000120913	399.0836078
SMIM10L2A	ENSG00000178947	134.1170153
LYAR	ENSG00000145220	907.29317
NTSR1	ENSG00000101188	4660.148416
PRICKLE2	ENSG00000163637	97.45251966
PXN	ENSG00000089159	12799.29026
MSANTD3	ENSG00000066697	348.7082916
FERMT1	ENSG00000101311	11452.21004
KBTBD8	ENSG00000163376	95.78608646
KLF6	ENSG00000067082	5208.490501
TMEM154	ENSG00000170006	1664.369139
DUSP14	ENSG00000276023	556.8022189
HS3ST1	ENSG00000002587	3973.654407
RHOD	ENSG00000173156	1375.946447
LPAR1	ENSG00000198121	30.9696916
ST3GAL1	ENSG00000008513	3608.333467
CHAC1	ENSG00000128965	655.0858818
HS3ST3B1	ENSG00000125430	78.77900079
FLNB	ENSG00000136068	24523.84044
PIP4K2A	ENSG00000150867	845.9302904
ANXA5	ENSG00000164111	3113.946223
CSF2	ENSG00000164400	47.56075732
SAMD4A	ENSG00000020577	1165.566813
CGNL1	ENSG00000128849	236.6632037
ELK3	ENSG00000111145	1547.808302
RASA3	ENSG00000185989	530.0525193
SNX9	ENSG00000130340	2007.114473

RGS20	ENSG00000147509	12.33606467
NPC1	ENSG00000141458	2252.938954
MAK16	ENSG00000198042	528.9616688
IL1A	ENSG00000115008	50.70473925
B3GNT5	ENSG00000176597	2161.874277
CCL20	ENSG00000115009	311.3456663
C3orf52	ENSG00000114529	670.4656524
LANCL3	ENSG00000147036	74.07689285
MYL9	ENSG00000101335	16.07518664
MYADM	ENSG00000179820	1859.808163
KLF14	ENSG00000266265	15.07837532
RBPMS2	ENSG00000166831	13.84968398
SH3TC1	ENSG00000125089	494.4652487
SQLE	ENSG00000104549	1784.524839
SHANK2	ENSG00000162105	541.601705
ENTPD7	ENSG00000198018	156.5456248
MYH15	ENSG00000144821	213.4615438
CCND1	ENSG00000110092	10460.75234
EFNB2	ENSG00000125266	2008.367265
PHC2	ENSG00000134686	3686.630672
MOSPD1	ENSG00000101928	1471.14515
SFN	ENSG00000175793	2246.168744
TRIB3	ENSG00000101255	4003.426555
RHOF	ENSG00000139725	4632.015421
PLK2	ENSG00000145632	1801.648797
SMOX	ENSG00000088826	1559.666629
SLC12A4	ENSG00000124067	614.1227206
HYI	ENSG00000178922	260.9308096
TNFRSF12A	ENSG00000006327	6945.609868
TRPV3	ENSG00000167723	35.97272679
NEK6	ENSG00000119408	462.7404432
ARHGAP40	ENSG00000124143	16.60424145
EHD4	ENSG00000103966	2398.418437
SERPINB8	ENSG00000166401	1004.488475
ZNF365	ENSG00000138311	14.10094341
TGM2	ENSG00000198959	1491.298841
UAP1	ENSG00000117143	1069.131776
DDX21	ENSG00000165732	5677.916418
NAMPT	ENSG00000105835	7417.979811
GREB1L	ENSG00000141449	1583.668412
SBDS	ENSG00000126524	3454.616995
OXTR	ENSG00000180914	1932.366387
CSGALNACT2	ENSG00000169826	70.4388718
AP1S2	ENSG00000182287	1293.319896

WWTR1	ENSG00000018408	2015.946076
CDCP1	ENSG00000163814	6253.830403
DDX60L	ENSG00000181381	2294.950055
FA2H	ENSG00000103089	837.0205805
EGFR	ENSG00000146648	7262.565176
IFRD1	ENSG00000006652	652.7201168
SLC20A1	ENSG00000144136	5517.588924
ELOVL1	ENSG00000066322	6205.333869
S100A14	ENSG00000189334	6523.819674
LIPG	ENSG00000101670	685.0779669
SEC14L2	ENSG00000100003	1194.586326
POLR3G	ENSG00000113356	677.1099404
CDC42EP2	ENSG00000149798	1374.158128
MAP1LC3B	ENSG00000140941	2657.841017
TRIM36	ENSG00000152503	419.3551332
YRDC	ENSG00000196449	619.0527847
LAT2	ENSG00000086730	616.9518234
KPNA4	ENSG00000186432	3303.711966
ACTG1	ENSG00000184009	81951.49596
ATG16L1	ENSG00000085978	2846.625263
P2RY2	ENSG00000175591	1307.048356
CORO1C	ENSG00000110880	5200.842418
P3H2	ENSG00000090530	67.3041676
TUBB2A	ENSG00000137267	649.4162649
ARHGEF2	ENSG00000116584	1330.094396
JUN	ENSG00000177606	744.5962565
NRG1	ENSG00000157168	12.5098715
PNO1	ENSG00000115946	1199.995692
C17orf51	ENSG00000212719	132.720553
DDIT4	ENSG00000168209	1973.384035
KCNK5	ENSG00000164626	1157.241706
KCTD5	ENSG00000167977	2079.229232
ZFP91-CNTF	ENSG00000255073	7.006222596
KRT16	ENSG00000186832	26.99475784
BMP2K	ENSG00000138756	673.7791603
CTNNAL1	ENSG00000119326	3206.840699
PDE4B	ENSG00000184588	127.519336
MAPKBP1	ENSG00000137802	696.4846718
LYST	ENSG00000143669	593.3486807
HOMER2	ENSG00000103942	315.2241263
NIFK	ENSG00000155438	2155.858637
LRCH1	ENSG00000136141	1022.101796
KLF2	ENSG00000127528	368.2654306
PAK1IP1	ENSG00000111845	871.1537465

ADAMTS16	ENSG00000145536	59.7701634
EIF5A2	ENSG00000163577	375.5138152
TNFRSF9	ENSG00000049249	13.03465307
MSANTD3-TMEFF1	ENSG00000251349	15.12471073
INHBA	ENSG00000122641	18.16717654
RABGEF1	ENSG00000154710	1231.351061
CFLAR	ENSG00000003402	2712.663559
ACSL4	ENSG00000068366	1356.599601
NPAS2	ENSG00000170485	661.4128406
ITCH	ENSG00000283472	1066.088038
TRMT6	ENSG00000089195	1527.63702
PROSER2	ENSG00000148426	1100.178678
TJP1	ENSG00000104067	4330.778543
SLC7A5	ENSG00000103257	13822.42403
ERCC1	ENSG00000012061	1876.290285
LHX5	ENSG00000089116	6.392985482
IL4R	ENSG00000077238	2449.860629
ASAP1	ENSG00000153317	1754.870002
CCNG2	ENSG00000138764	1930.228001
LIF	ENSG00000128342	2189.43959
SERINC5	ENSG00000164300	3111.745094
SGK2	ENSG00000101049	853.6164766
PHF21A	ENSG00000135365	280.8881998
FAM227A	ENSG00000184949	32.08428218
JAKMIP3	ENSG00000188385	11.44921946
LRRC6	ENSG00000129295	79.22310808
RAB3D	ENSG00000105514	393.9075477
GRAMD2	ENSG00000175318	15.08588608
PATL2	ENSG00000229474	7.524476848
OVOL2	ENSG00000125850	67.75522445
COQ8A	ENSG00000163050	274.6952163
FLT3LG	ENSG00000090554	24.87384889
LGR4	ENSG00000205213	1423.777951
PTPN6	ENSG00000111679	473.5205144
SOX12	ENSG00000177732	331.7056126
SGPP2	ENSG00000163082	496.4117923
MLLT3	ENSG00000171843	240.9412028
SEC31B	ENSG00000075826	60.18439801
ZFPM1	ENSG00000179588	95.44217923
PLCL1	ENSG00000115896	6.211431355
THAP8	ENSG00000161277	31.05249321
ANKRD9	ENSG00000156381	826.0966315
ADCY4	ENSG00000129467	37.91935153
FBXL20	ENSG00000108306	205.4636819

GDPD1	ENSG00000153982	28.96838631
NAPSA	ENSG00000131400	18.26471626
SLC11A2	ENSG00000110911	1548.069284
SPHK2	ENSG00000063176	587.6936886
CDK19	ENSG00000155111	269.9599784
EPHB3	ENSG00000182580	281.3963933
SP5	ENSG00000204335	38.06876306
WDR31	ENSG00000148225	62.86772272
PDX1	ENSG00000139515	111.1858337
SLC2A10	ENSG00000197496	390.9510163
LRRC56	ENSG00000161328	59.51552969
CH507-9B2.1	ENSG00000277117	160.4426057
MLXIPL	ENSG00000009950	199.7743181
ACPP	ENSG00000014257	7.447552767
ARID5B	ENSG00000150347	204.7271237
C1orf21	ENSG00000116667	33.61762527
LRRC26	ENSG00000184709	15.44689385
ANK1	ENSG00000029534	28.09187701
GGT6	ENSG00000167741	19.96278364
ACCS	ENSG00000110455	132.4055706
ACBD4	ENSG00000181513	206.3526472
IFT74	ENSG00000096872	90.04855426
SPTLC3	ENSG00000172296	70.0035204
GOLPH3L	ENSG00000143457	538.8374024
LGR5	ENSG00000139292	8.629157212
STPG3	ENSG00000197768	6.233384416
NUDT6	ENSG00000170917	54.5329536
CEP162	ENSG00000135315	77.65504982
PTGER4	ENSG00000171522	35.65769353
SEMA4G	ENSG00000095539	1767.910131
LIN7B	ENSG00000104863	41.83456046
BRCA1	ENSG00000012048	521.1085495
PIP5K1B	ENSG00000107242	389.2059555
KCNH3	ENSG00000135519	30.41971505
FSBP	ENSG00000265817	32.52780998
PITX2	ENSG00000164093	68.1597601
SARM1	ENSG00000004139	74.66331522
TRIM38	ENSG00000112343	592.4050109
PPP1R32	ENSG00000162148	13.62606865
ORAI3	ENSG00000175938	136.9965923
HDHD3	ENSG00000119431	923.6205588
FOXD2	ENSG00000186564	83.70509581
SLFN13	ENSG00000154760	690.2094395
BEND7	ENSG00000165626	10.28118097

PXMP4	ENSG00000101417	296.4269542
BAHCC1	ENSG00000266074	311.3156918
HOXC4	ENSG00000198353	137.0014039
RPRM	ENSG00000177519	22.79694953
ORC1	ENSG00000085840	273.5917814
PRRT2	ENSG00000167371	64.24454038
FOXJ2	ENSG00000065970	266.4552326
MNS1	ENSG00000138587	43.77686618
CHST12	ENSG00000136213	138.6986219
SCX	ENSG00000260428	47.03677245
BBX	ENSG00000114439	733.9232719
TBX3	ENSG00000135111	12.11499746
SNAI3	ENSG00000185669	37.02386044
ANKRA2	ENSG00000164331	227.3884068
ARHGEF37	ENSG00000183111	126.2934683
USP28	ENSG00000048028	331.9086652
FAM229A	ENSG00000225828	41.17902151
GLS2	ENSG00000135423	38.04273671
FZD4	ENSG00000174804	53.00945373
PID1	ENSG00000153823	57.9933645
ZFP14	ENSG00000142065	32.89927598
RASSF9	ENSG00000198774	51.10382535
PLA2G6	ENSG00000184381	92.18414084
CABLES2	ENSG00000149679	100.54562
GRAMD1B	ENSG00000023171	2939.580299
ALAD	ENSG00000148218	815.6380906
TRIM68	ENSG00000167333	147.3597602
MSRB1	ENSG00000198736	389.0821823
CA3	ENSG00000164879	11.72442745
RASSF4	ENSG00000107551	7.196241304
GAL3ST1	ENSG00000128242	275.8938573
DPYSL2	ENSG00000092964	493.8921445
RP11-111M22.2	ENSG00000179240	23.24159756
KLHDC2	ENSG00000165516	852.203291
EBF4	ENSG00000088881	10.64473595
CARNS1	ENSG00000172508	125.9064539
SHROOM1	ENSG00000164403	1104.291621
SSBP3	ENSG00000157216	362.054416
TLR3	ENSG00000164342	49.38475172
ANG	ENSG00000214274	86.0953223
CREB3L1	ENSG00000157613	911.5537601
APOBEC3D	ENSG00000243811	28.90082561
BRIP1	ENSG00000136492	203.0100577
ZMYM3	ENSG00000147130	654.768719

MKRN2OS	ENSG00000225526	53.99459562
VNN1	ENSG00000112299	248.2647269
ZSWIM5	ENSG00000162415	68.93932329
BTN3A2	ENSG00000186470	454.7534653
ARRB1	ENSG00000137486	500.5678384
ZDHHC1	ENSG00000159714	30.91855461
AZIN2	ENSG00000142920	19.41810547
KLHL22	ENSG00000099910	49.76031931
SCAMP5	ENSG00000198794	43.32996524
ST5	ENSG00000166444	297.8994043
RAB40C	ENSG00000197562	394.8337521
MOB3B	ENSG00000120162	48.39915954
CITED1	ENSG00000125931	193.5671225
BTG2	ENSG00000159388	91.04046337
GRB7	ENSG00000141738	557.1339364
CREB3L4	ENSG00000143578	272.7148787
LRRC75B	ENSG00000178026	161.7126326
CCDC103	ENSG00000167131	14.99612814
WDR72	ENSG00000166415	170.9885423
AMY2B	ENSG00000240038	15.38760009
RORC	ENSG00000143365	14.43595454
TRANK1	ENSG00000168016	339.1118628
RAD54B	ENSG00000283460	80.38109872
MGAT3	ENSG00000128268	259.5609523
KHK	ENSG00000138030	177.9343782
PRRT1	ENSG00000204314	31.55679479
TTLL6	ENSG00000170703	7.841468648
TNFRSF11A	ENSG00000141655	145.5541491
UNC5B	ENSG00000107731	372.9039419
PASK	ENSG00000115687	281.0331862
PLSCR4	ENSG00000114698	50.57477991
PIK3R3	ENSG00000117461	246.5745598
FMO5	ENSG00000131781	49.71970801
INSR	ENSG00000171105	271.1904198
RNFT2	ENSG00000135119	42.4141651
APOL2	ENSG00000128335	1255.248442
PTPN13	ENSG00000163629	1432.428019
BHLHE41	ENSG00000123095	1986.188931
PROCA1	ENSG00000167525	55.30643623
IGFLR1	ENSG00000126246	24.01123679
DOCK11	ENSG00000147251	69.81380362
PPP1R1B	ENSG00000131771	319.672398
TRIM34	ENSG00000258659	190.0171848
SLCO2B1	ENSG00000137491	42.20185761

DISP1	ENSG00000154309	32.71626619
TJP3	ENSG00000105289	1682.813627
C20orf96	ENSG00000196476	167.6984847
PTCH1	ENSG00000185920	101.9192777
GUCY2C	ENSG00000070019	166.1788128
PPFIBP2	ENSG00000166387	390.0176703
BBC3	ENSG00000105327	219.1167931
NMNAT3	ENSG00000163864	58.08970524
PLEKHH3	ENSG00000068137	270.3353343
NYNRIN	ENSG00000205978	107.2222572
IGSF9	ENSG00000085552	304.6390224
BTBD8	ENSG00000189195	26.61939334
HRCT1	ENSG00000196196	48.59286746
ABHD4	ENSG00000100439	683.4966606
CAPN5	ENSG00000149260	2396.751486
CROT	ENSG00000005469	121.4810881
DEPTOR	ENSG00000155792	22.01620096
HNF4G	ENSG00000164749	594.0527543
CAPN3	ENSG00000092529	9.385174927
CASZ1	ENSG00000130940	289.3682912
PIWIL2	ENSG00000197181	8.25968078
FOXN3	ENSG00000053254	278.5396266
TRIM31	ENSG00000204616	3763.995009
RARB	ENSG00000077092	76.661419
SLC7A2	ENSG00000003989	99.95105496
KIAA1683	ENSG00000130518	69.15553347
E2F8	ENSG00000129173	155.6987862
TOX3	ENSG00000103460	257.3824292
TSHZ2	ENSG00000182463	14.70423904
NOXO1	ENSG00000196408	9.447223811
ARHGEF40	ENSG00000165801	122.1949689
CDC25C	ENSG00000158402	401.5264189
CCDC183	ENSG00000213213	74.26448703
SEMA5A	ENSG00000112902	276.5874016
BTN2A2	ENSG00000124508	59.93937991
CNTNAP1	ENSG00000108797	91.44861314
SEC16B	ENSG00000120341	24.39263711
TESK2	ENSG00000070759	42.54439169
ZNF618	ENSG00000157657	5.446962535
SOWAHD	ENSG00000187808	7.487047052
C10orf10	ENSG00000165507	23.41606959
GPR19	ENSG00000183150	14.78001964
CAPS	ENSG00000105519	53.60681587
PRR15	ENSG00000176532	1067.177256

YPEL3	ENSG00000090238	331.4810341
NEURL1B	ENSG00000214357	15.79541681
TTC30B	ENSG00000196659	113.8372912
LRRC45	ENSG00000169683	359.5971649
EXOC3L4	ENSG00000205436	80.10157556
ICOSLG	ENSG00000160223	159.1941201
PLCD1	ENSG00000187091	92.99546282
NR0B2	ENSG00000131910	9.244185644
ID3	ENSG00000117318	2619.416619
ARFGEF3	ENSG00000112379	529.7157356
SOX2	ENSG00000181449	19.85611774
MYLIP	ENSG00000007944	174.5544958
BCL2L14	ENSG00000121380	175.247764
HSD11B2	ENSG00000176387	624.0536925
PDZD3	ENSG00000172367	140.5527899
ABCB6	ENSG00000115657	200.2374374
PDK2	ENSG00000005882	242.4350269
RAB37	ENSG00000172794	68.510462
FAM214A	ENSG00000047346	235.1366927
C2orf15	ENSG00000273045	87.46860411
ENGASE	ENSG00000167280	776.1611176
HR	ENSG00000168453	389.9178347
ILDR1	ENSG00000145103	138.0588167
TSPAN12	ENSG00000106025	14.63994863
FAM117A	ENSG00000121104	66.13338856
FREM2	ENSG00000150893	800.3938239
NDRG2	ENSG00000165795	102.8578529
TRIM46	ENSG00000163462	18.0131413
GPRC5C	ENSG00000170412	2140.878523
KCNC4	ENSG00000116396	781.8815256
SLC2A12	ENSG00000146411	291.3086648
ZNF488	ENSG00000265763	121.0530532
CLRN3	ENSG00000180745	345.6277051
PEX5L	ENSG00000114757	65.91454377
PRICKLE4	ENSG00000278224	21.26516002
LCN12	ENSG00000184925	46.11548837
EXOC3L1	ENSG00000179044	9.126466677
C6orf222	ENSG00000189325	140.7757985
PPP1R3E	ENSG00000235194	211.7505825
RHOV	ENSG00000104140	65.87005407
CLDN3	ENSG00000165215	651.3157918
ST3GAL5	ENSG00000115525	475.9072853
HEXIM2	ENSG00000168517	96.88855474
EML1	ENSG00000066629	29.73305324

PPM1H	ENSG00000111110	34.25680883
ALPK1	ENSG00000073331	275.4964286
TBC1D29	ENSG00000266733	8.725404755
ABCC6	ENSG00000091262	179.1496836
BCAS1	ENSG00000064787	157.5355819
GATSL2	ENSG00000274070	22.75607292
SSTR5	ENSG00000162009	38.584264
NSUN7	ENSG00000179299	151.1379175
DOC2A	ENSG00000149927	225.5925501
C2orf88	ENSG00000187699	280.5311117
SDPR	ENSG00000168497	89.6720907
CLEC2D	ENSG00000069493	9.918111967
ERBB2	ENSG00000141736	2506.285615
SOWAHB	ENSG00000186212	117.0082435
TRIM62	ENSG00000116525	98.82958815
ELF3	ENSG00000163435	4538.920916
SLC25A27	ENSG00000153291	13.95913205
HOXA13	ENSG00000106031	11.20580557
CTD-2132N18.2	ENSG00000267221	28.18590992
CHN2	ENSG00000106069	95.02315051
DUSP19	ENSG00000162999	32.07700034
RALGPS1	ENSG00000136828	122.2571013
C2orf81	ENSG00000159239	15.67583007
AQP7	ENSG00000165269	40.4133497
PLA2G4F	ENSG00000168907	119.7633004
KCNE3	ENSG00000175538	480.1524718
CHDH	ENSG00000016391	49.44595981
IRF1	ENSG00000125347	965.3484939
PDE5A	ENSG00000138735	63.84175112
KCNS3	ENSG00000170745	783.4578133
MAP3K8	ENSG00000107968	32.63104193
NFATC4	ENSG00000100968	41.73649533
MXD3	ENSG00000213347	197.0872699
SUOX	ENSG00000139531	559.0814625
FZD2	ENSG00000180340	11.85520583
ALDH3B2	ENSG00000132746	29.16046989
CYP24A1	ENSG00000019186	5443.12781
TRIM2	ENSG00000109654	851.1226559
RASA4	ENSG00000105808	451.2031349
KLC4	ENSG00000137171	309.028255
KCNMB4	ENSG00000135643	136.9010401
CBX7	ENSG00000100307	160.3095243
ZNF608	ENSG00000168916	367.2608835
LDLRAD1	ENSG00000203985	72.61232291

STAG3	ENSG00000066923	82.2833682
PLEKHG7	ENSG00000187510	10.73810932
BMF	ENSG00000104081	99.91728143
LNX1	ENSG00000072201	37.67964566
ANKS4B	ENSG00000175311	402.8920976
C21orf58	ENSG00000160298	192.7083139
CACNA1I	ENSG00000100346	20.09599706
PIK3IP1	ENSG00000100100	99.05774959
GBP4	ENSG00000162654	81.07679353
DEGS2	ENSG00000168350	115.0047545
KCNK10	ENSG00000100433	9.608899503
GLYCTK	ENSG00000168237	919.7772908
ERBB3	ENSG00000065361	5378.559099
APOL3	ENSG00000128284	264.5944385
FZD7	ENSG00000155760	575.9544997
TNFSF10	ENSG00000121858	406.7155802
DAPK1	ENSG00000196730	781.4701946
RHOU	ENSG00000116574	555.2231212
TP73	ENSG00000078900	138.0867906
FRAT2	ENSG00000181274	273.1617933
FFAR4	ENSG00000186188	174.0344197
PROC	ENSG00000115718	67.98382283
BCO1	ENSG00000135697	37.5539518
PPARGC1A	ENSG00000109819	149.9619836
PKDCC	ENSG00000162878	2641.850414
KBTBD3	ENSG00000182359	55.23943015
LRRC31	ENSG00000114248	201.0959121
C5AR1	ENSG00000197405	71.83312982
GATS	ENSG00000239521	133.9121644
ATP10B	ENSG00000118322	1628.779873
PAQR8	ENSG00000170915	242.1213395
VIPR1	ENSG00000114812	461.6316354
C5orf56	ENSG00000197536	90.80615189
CLDN2	ENSG00000165376	234.2487486
GRHL3	ENSG00000158055	221.2708392
LMO4	ENSG00000143013	1196.191842
ICK	ENSG00000112144	145.3802007
SOWAHA	ENSG00000198944	78.21828444
IHH	ENSG00000163501	70.80393156
RTP4	ENSG00000136514	186.3445743
NCMAP	ENSG00000184454	49.76729578
GAS2	ENSG00000148935	45.27039512
ISX	ENSG00000175329	534.4747833
HOXC5	ENSG00000172789	24.9719122

ACER2	ENSG00000177076	165.27754
SPDEF	ENSG00000124664	123.3134376
SMPD3	ENSG00000103056	83.73718684
CDX2	ENSG00000165556	1907.780712
MAP2K6	ENSG00000108984	65.0916883
MISP3	ENSG00000141854	22.79634986
HOXC6	ENSG00000197757	198.3516719
DAPK2	ENSG00000035664	158.812956
PALM3	ENSG00000187867	17.87833648
RAB26	ENSG00000167964	37.3632844
CDKN2C	ENSG00000123080	44.47732431
E2F2	ENSG00000007968	151.5507942
BTN3A1	ENSG00000026950	335.4609845
PRICKLE4	ENSG00000124593	436.5899583
PDE7B	ENSG00000171408	27.59994335
AGFG2	ENSG00000106351	697.8575174
INPP5J	ENSG00000185133	41.37618067
BTN3A3	ENSG00000111801	190.2556901
ZNF362	ENSG00000160094	53.43160443
MEF2C	ENSG00000081189	304.3735939
TLX1	ENSG00000107807	34.40982645
DMBX1	ENSG00000197587	24.92774122
PRR15L	ENSG00000167183	585.3268037
RAB40B	ENSG00000141542	128.4781671
SESN3	ENSG00000149212	400.2749986
YPEL2	ENSG00000175155	70.28954384
PDK4	ENSG00000004799	147.0177739
ADRA2A	ENSG00000150594	27.84999568
SLC40A1	ENSG00000138449	813.0760682
GNG2	ENSG00000186469	138.6090335
CYP1B1	ENSG00000138061	42.30332468
GNA14	ENSG00000156049	11.90996735
KIAA1211L	ENSG00000196872	126.1463918
FRMPD3	ENSG00000147234	29.68141814
SAMD11	ENSG00000187634	66.50403882
NUAK2	ENSG00000163545	133.6918041
LRRC66	ENSG00000188993	204.2644233
CYP26B1	ENSG00000003137	55.7819498
C9orf152	ENSG00000188959	33.56320943
SLAIN1	ENSG00000139737	30.60492599
GRAMD1C	ENSG00000178075	269.7605232
C2orf72	ENSG00000204128	98.65463679
PBX1	ENSG00000185630	169.7519472
DBP	ENSG00000105516	47.99590531

ACSS1	ENSG00000154930	236.6628752
KLHDC7A	ENSG00000179023	48.74114706
ASAP3	ENSG00000088280	86.7590736
EPN3	ENSG00000049283	306.6832384
FRY	ENSG00000073910	30.96663461
SYNM	ENSG00000182253	31.01431547
TMEM37	ENSG00000171227	294.8711391
ACACB	ENSG00000076555	34.32954862
VWA5B2	ENSG00000145198	44.48293626
CYP1A1	ENSG00000140465	88.98951101
FRAT1	ENSG00000165879	64.90632562
RASL11A	ENSG00000122035	601.7090523
B4GAT1	ENSG00000174684	251.8768559
IKZF2	ENSG00000030419	143.3034696
PLCH1	ENSG00000114805	466.1806432
COLCA2	ENSG00000214290	138.3116002
RBBP8NL	ENSG00000130701	95.28430461

reated HPAF-II cells)

log2FoldChange	pvalue	padj
4.02771941	2.55E-44	2.23E-42
3.299231627	1.21E-156	8.21E-153
3.289778789	5.56E-23	1.39E-21
3.280832516	5.87E-24	1.58E-22
3.046019114	2.28E-37	1.38E-35
2.916213064	1.14E-54	1.61E-52
2.86543815	1.57E-197	2.14E-193
2.8065014	7.15E-28	2.56E-26
2.755852709	2.04E-30	8.53E-29
2.70806329	1.13E-105	1.28E-102
2.458530873	8.26E-46	8.03E-44
2.433709815	1.02E-34	5.33E-33
2.429118482	1.03E-65	2.69E-63
2.383384056	2.02E-36	1.15E-34
2.377383522	9.62E-62	2.08E-59
2.312983212	1.39E-145	6.33E-142
2.27958824	1.12E-76	4.22E-74
2.279055462	1.98E-102	1.80E-99
2.207871171	3.02E-42	2.30E-40
2.203203571	2.89E-19	5.30E-18
2.188201967	7.41E-13	7.79E-12
2.159955576	7.63E-43	6.18E-41
2.146154285	5.69E-14	6.69E-13
2.131968219	2.44E-16	3.57E-15
2.131115241	1.84E-31	8.24E-30
2.11892528	5.75E-26	1.77E-24
2.104131819	6.59E-92	4.98E-89
2.091806875	3.64E-55	5.21E-53
2.08706946	2.31E-38	1.48E-36
2.075988065	1.20E-66	3.26E-64
2.066054643	7.85E-43	6.32E-41
2.04696375	7.40E-145	2.52E-141
2.035555805	3.46E-75	1.18E-72
2.032251795	7.57E-09	5.00E-08
2.029691385	3.21E-101	2.73E-98
2.029184289	7.19E-67	2.04E-64
2.025550417	3.09E-48	3.34E-46
2.025265267	5.11E-134	9.94E-131
2.019896137	1.02E-42	8.17E-41
2.019720057	5.37E-23	1.35E-21
2.018142064	5.79E-57	9.16E-55

2.014330754	9.11E-100	7.29E-97
2.013210621	2.24E-119	3.39E-116
1.996670506	4.73E-15	6.14E-14
1.990007958	7.65E-67	2.13E-64
1.9814951	5.95E-16	8.31E-15
1.973751828	2.41E-39	1.61E-37
1.968883834	9.43E-117	1.28E-113
1.968142398	9.79E-33	4.57E-31
1.958892394	8.40E-09	5.52E-08
1.95225685	1.07E-09	7.89E-09
1.947135597	4.97E-10	3.84E-09
1.938450387	9.26E-19	1.65E-17
1.885449802	7.35E-18	1.21E-16
1.883766359	3.91E-36	2.22E-34
1.872828263	8.61E-89	5.33E-86
1.865869808	7.71E-92	5.53E-89
1.857769507	1.34E-24	3.72E-23
1.853563241	8.71E-20	1.67E-18
1.851735682	2.14E-08	1.34E-07
1.845042871	1.88E-51	2.30E-49
1.841594569	4.55E-23	1.15E-21
1.838300379	2.01E-87	1.14E-84
1.826820521	1.62E-30	6.87E-29
1.823846213	2.65E-13	2.91E-12
1.823642853	2.20E-89	1.43E-86
1.813760371	2.44E-24	6.71E-23
1.806583284	1.75E-50	2.05E-48
1.795206952	1.42E-10	1.17E-09
1.786778465	6.91E-08	4.02E-07
1.774713416	9.71E-86	5.29E-83
1.772347788	2.09E-120	3.56E-117
1.758641851	6.22E-10	4.73E-09
1.75478663	8.78E-82	3.73E-79
1.753292822	1.19E-13	1.36E-12
1.751565164	5.57E-135	1.26E-131
1.741503763	1.58E-63	3.71E-61
1.740300171	4.75E-42	3.55E-40
1.733115067	8.53E-28	3.04E-26
1.723162561	5.45E-09	3.66E-08
1.716194726	1.14E-58	2.12E-56
1.710380843	1.84E-110	2.27E-107
1.704987671	1.51E-49	1.70E-47
1.702061312	7.30E-74	2.37E-71
1.69895098	1.14E-104	1.19E-101

1.693268495	4.31E-28	1.57E-26
1.692514148	1.43E-57	2.38E-55
1.691123159	1.18E-78	4.59E-76
1.690745428	3.08E-28	1.14E-26
1.68103749	5.93E-138	1.62E-134
1.671774483	1.83E-88	1.08E-85
1.671563952	3.12E-59	6.16E-57
1.670770415	1.39E-102	1.35E-99
1.662874855	6.44E-72	1.99E-69
1.66171829	5.73E-07	2.92E-06
1.66105028	5.29E-34	2.65E-32
1.658617896	2.17E-76	7.78E-74
1.647529863	4.71E-45	4.28E-43
1.642494423	2.88E-39	1.90E-37
1.639013183	3.70E-09	2.54E-08
1.624401453	1.41E-19	2.67E-18
1.615586725	9.64E-18	1.57E-16
1.612438373	1.26E-17	2.03E-16
1.609412298	5.47E-20	1.07E-18
1.602474906	3.60E-20	7.19E-19
1.597727858	7.01E-59	1.34E-56
1.591574199	1.27E-17	2.03E-16
1.580808271	2.54E-18	4.38E-17
1.563390139	1.51E-75	5.27E-73
1.560842796	9.86E-21	2.04E-19
1.560501856	2.15E-29	8.36E-28
1.547528744	6.25E-35	3.31E-33
1.538951212	7.96E-44	6.73E-42
1.535697943	1.53E-06	7.26E-06
1.53302345	1.06E-45	1.01E-43
1.526833013	4.87E-43	4.00E-41
1.524944302	8.14E-20	1.56E-18
1.523723899	1.85E-19	3.47E-18
1.514884339	9.21E-38	5.73E-36
1.510750065	2.39E-64	5.81E-62
1.503523544	1.05E-83	4.76E-81
1.500390302	5.52E-65	1.39E-62
1.490150148	7.63E-53	1.01E-50
1.489909204	1.20E-18	2.12E-17
1.488429229	2.73E-85	1.38E-82
1.485210947	4.96E-42	3.69E-40
1.484854198	4.02E-58	7.10E-56
1.48362765	2.06E-69	6.11E-67
1.479398196	6.83E-20	1.32E-18

1.476536219	5.12E-05	0.00018951
1.47393096	1.26E-19	2.40E-18
1.472128414	3.00E-26	9.60E-25
1.471980764	5.75E-51	6.81E-49
1.467129216	2.46E-42	1.90E-40
1.463954095	1.25E-61	2.66E-59
1.462124384	3.43E-85	1.67E-82
1.45819697	1.39E-85	7.27E-83
1.45567465	1.95E-05	7.77E-05
1.453438958	1.72E-42	1.34E-40
1.449396519	5.15E-41	3.63E-39
1.447088427	7.22E-45	6.43E-43
1.443561698	1.78E-83	7.83E-81
1.441852251	1.83E-56	2.80E-54
1.44140293	4.47E-51	5.34E-49
1.440933004	2.72E-14	3.30E-13
1.436945924	1.75E-05	7.04E-05
1.428536236	3.98E-12	3.87E-11
1.420751331	5.83E-79	2.33E-76
1.412309949	1.15E-76	4.24E-74
1.412088715	2.11E-18	3.66E-17
1.407166699	1.34E-17	2.14E-16
1.403962839	4.04E-56	6.10E-54
1.401479577	5.05E-53	6.81E-51
1.397306425	1.03E-05	4.30E-05
1.388790263	1.24E-62	2.76E-60
1.38655977	9.11E-36	5.04E-34
1.385315027	1.56E-33	7.46E-32
1.377074793	1.52E-62	3.34E-60
1.373352606	1.32E-36	7.63E-35
1.37331585	7.10E-81	2.93E-78
1.367178307	0.00018039	0.00060016
1.360902432	5.38E-58	9.39E-56
1.359863544	8.12E-55	1.15E-52
1.35579167	1.73E-43	1.44E-41
1.352848683	4.54E-56	6.71E-54
1.352502056	2.36E-28	8.76E-27
1.348721709	6.41E-57	1.00E-54
1.34851504	5.08E-26	1.58E-24
1.348369709	9.47E-06	3.96E-05
1.347792064	2.33E-51	2.81E-49
1.339320466	4.42E-06	1.95E-05
1.33887163	2.10E-60	4.33E-58
1.337179383	1.67E-11	1.52E-10

1.33457192	5.23E-91	3.56E-88
1.331914979	5.73E-84	2.69E-81
1.329431543	1.55E-52	1.99E-50
1.329166622	1.26E-06	6.06E-06
1.321428119	4.28E-46	4.22E-44
1.321291006	4.15E-41	2.96E-39
1.311948891	3.97E-41	2.84E-39
1.305086038	7.46E-21	1.56E-19
1.303871877	4.53E-30	1.86E-28
1.303098164	5.16E-09	3.48E-08
1.296818843	5.67E-16	7.96E-15
1.296486234	1.67E-07	9.18E-07
1.295928976	3.50E-05	0.00013359
1.286656487	3.44E-28	1.26E-26
1.284467518	4.28E-45	3.91E-43
1.283490626	4.33E-29	1.67E-27
1.283068588	4.81E-07	2.48E-06
1.279751764	4.28E-37	2.54E-35
1.272546885	3.18E-57	5.15E-55
1.270887187	0.00049853	0.0015185
1.268273916	2.95E-09	2.05E-08
1.266464051	0.00050887	0.00154499
1.265715256	5.83E-11	4.97E-10
1.263953868	8.69E-58	1.48E-55
1.26229169	2.53E-30	1.05E-28
1.25807023	1.22E-17	1.97E-16
1.256239662	3.31E-11	2.91E-10
1.255552795	4.22E-33	1.99E-31
1.254492904	2.40E-18	4.14E-17
1.252688401	1.32E-07	7.35E-07
1.250935273	5.70E-25	1.63E-23
1.248466939	8.78E-21	1.82E-19
1.247777721	1.88E-33	8.97E-32
1.245238295	1.14E-34	5.96E-33
1.245206272	1.21E-58	2.22E-56
1.243548196	6.42E-48	6.83E-46
1.240887066	0.00065044	0.00192836
1.240563543	1.30E-47	1.36E-45
1.239848021	1.53E-05	6.18E-05
1.235979536	1.04E-23	2.73E-22
1.235712777	1.33E-22	3.22E-21
1.232282266	0.00045876	0.0014076
1.228613317	6.68E-06	2.86E-05
1.227204377	4.42E-35	2.37E-33

1.225795274	1.62E-06	7.65E-06
1.223587073	1.93E-50	2.25E-48
1.222074404	2.05E-39	1.38E-37
1.222071374	1.55E-51	1.92E-49
1.215696237	1.62E-34	8.34E-33
1.213676569	2.18E-65	5.60E-63
1.212343041	4.05E-43	3.34E-41
1.211968015	2.67E-49	2.96E-47
1.211681136	2.43E-49	2.71E-47
1.210306754	1.05E-52	1.36E-50
1.20847629	1.17E-57	1.96E-55
1.2057431	0.00060241	0.00180128
1.20432585	2.86E-48	3.12E-46
1.204203284	8.90E-05	0.00031559
1.204147656	1.13E-45	1.08E-43
1.203512813	1.38E-19	2.61E-18
1.200468027	8.91E-27	2.97E-25
1.198094477	8.12E-21	1.69E-19
1.19539285	7.81E-09	5.15E-08
1.188302735	1.85E-46	1.85E-44
1.188054728	4.33E-56	6.48E-54
1.186124671	5.06E-09	3.42E-08
1.184197556	2.06E-64	5.10E-62
1.183886186	1.01E-24	2.83E-23
1.18332591	4.60E-75	1.53E-72
1.182242501	3.37E-09	2.33E-08
1.182062349	4.18E-44	3.55E-42
1.175895732	1.24E-42	9.77E-41
1.175344308	4.45E-27	1.53E-25
1.173371536	1.44E-49	1.64E-47
1.172715851	8.72E-42	6.45E-40
1.171314961	4.73E-05	0.0001765
1.169272325	2.34E-72	7.42E-70
1.165963881	6.04E-18	1.00E-16
1.165239866	5.19E-09	3.50E-08
1.164332071	4.71E-46	4.61E-44
1.1623793	1.44E-29	5.66E-28
1.162135478	1.75E-66	4.66E-64
1.160371564	3.41E-06	1.54E-05
1.156221495	2.05E-52	2.59E-50
1.151592829	9.38E-15	1.19E-13
1.151355999	3.06E-44	2.65E-42
1.148338247	1.07E-27	3.79E-26
1.148314664	4.44E-35	2.37E-33

1.144820557	0.0010832	0.00305378
1.141246622	1.42E-59	2.83E-57
1.140687538	4.64E-34	2.34E-32
1.139512892	2.81E-06	1.29E-05
1.134846472	9.58E-28	3.40E-26
1.134395347	3.24E-13	3.53E-12
1.132503867	2.30E-37	1.39E-35
1.131563023	2.94E-08	1.80E-07
1.130743217	0.00080348	0.00233083
1.126142349	2.71E-29	1.05E-27
1.124812249	0.00087371	0.00250732
1.124004986	0.00115863	0.00324892
1.123758395	7.09E-25	2.02E-23
1.120135036	3.43E-57	5.49E-55
1.119856821	4.62E-29	1.77E-27
1.119467354	6.25E-13	6.63E-12
1.116493684	9.44E-15	1.20E-13
1.113321257	5.67E-58	9.78E-56
1.112327896	2.69E-57	4.41E-55
1.111412754	9.12E-70	2.76E-67
1.111118669	2.59E-28	9.59E-27
1.110630945	1.04E-42	8.25E-41
1.107870318	1.93E-12	1.94E-11
1.107410544	3.98E-39	2.62E-37
1.104905665	2.75E-37	1.64E-35
1.10407426	1.93E-45	1.81E-43
1.103438977	6.50E-22	1.48E-20
1.10117233	1.17E-16	1.75E-15
1.100664251	3.00E-58	5.44E-56
1.098753739	0.00014121	0.00048182
1.098623898	5.97E-27	2.03E-25
1.096350643	0.00091641	0.00261772
1.096283268	6.59E-56	9.65E-54
1.095297023	8.67E-37	5.08E-35
1.093650431	0.00206441	0.00549459
1.092642859	1.04E-09	7.64E-09
1.092469014	4.81E-39	3.15E-37
1.0919704	5.63E-63	1.28E-60
1.091165954	1.62E-37	9.91E-36
1.090074227	3.34E-40	2.29E-38
1.088874595	1.02E-58	1.92E-56
1.088628051	4.83E-41	3.42E-39
1.085021534	3.14E-07	1.66E-06
1.084462477	4.92E-23	1.24E-21

1.082937981	6.42E-48	6.83E-46
1.081279611	3.56E-61	7.47E-59
1.079874882	9.04E-36	5.02E-34
1.078044385	1.08E-31	4.83E-30
1.077507826	1.99E-63	4.59E-61
1.075413583	3.26E-23	8.32E-22
1.074974649	1.13E-46	1.14E-44
1.074251876	8.12E-53	1.06E-50
1.072690915	2.92E-46	2.90E-44
1.071001002	2.54E-27	8.88E-26
1.070617294	5.84E-39	3.80E-37
1.06815309	2.57E-18	4.41E-17
1.06760934	8.91E-25	2.52E-23
1.064529855	6.88E-30	2.78E-28
1.063705759	5.67E-20	1.10E-18
1.060967366	3.03E-30	1.25E-28
1.060014103	9.95E-29	3.74E-27
1.055537102	3.19E-37	1.90E-35
1.054184595	2.41E-11	2.15E-10
1.052979256	7.66E-54	1.06E-51
1.049648692	1.71E-26	5.58E-25
1.047454212	1.39E-49	1.59E-47
1.045428884	2.88E-06	1.31E-05
1.041724471	4.17E-23	1.06E-21
1.041296312	6.40E-34	3.16E-32
1.040668483	1.05E-33	5.09E-32
1.040545647	0.00282954	0.0072902
1.040446272	8.96E-40	6.04E-38
1.040141851	5.32E-08	3.16E-07
1.036820949	5.18E-08	3.08E-07
1.034173746	7.75E-21	1.62E-19
1.032954386	1.02E-48	1.12E-46
1.032908802	0.00341222	0.00863348
1.032143367	0.00063622	0.00189115
1.03177058	1.77E-17	2.81E-16
1.029971506	1.07E-56	1.66E-54
1.02916338	3.80E-09	2.60E-08
1.028550396	6.79E-18	1.12E-16
1.023464027	8.44E-19	1.52E-17
1.022536187	1.40E-17	2.23E-16
1.021518386	4.70E-47	4.85E-45
1.020841399	2.85E-33	1.35E-31
1.020822661	6.31E-16	8.81E-15
1.020797086	1.35E-34	7.02E-33

1.02043466	4.27E-06	1.89E-05
1.019631258	3.98E-18	6.75E-17
1.017778554	0.00367344	0.00922527
1.014328968	0.00245154	0.00641833
1.014318227	0.00200349	0.00533767
1.013588706	8.50E-40	5.75E-38
1.012759729	7.13E-41	5.00E-39
1.011817358	9.51E-26	2.91E-24
1.011196257	2.17E-23	5.58E-22
1.01116606	5.72E-31	2.47E-29
1.010001712	9.69E-34	4.71E-32
1.005943628	9.02E-30	3.63E-28
1.005483811	3.43E-32	1.56E-30
1.005086195	2.15E-40	1.48E-38
1.003449968	6.80E-34	3.33E-32
1.002945299	0.00593212	0.01421328
1.002763553	6.03E-45	5.44E-43
1.002237955	5.64E-47	5.77E-45
1.002216172	5.89E-08	3.47E-07
1.001156396	1.11E-41	8.12E-40
-1.000061493	2.16E-31	9.59E-30
-1.000247455	3.81E-26	1.21E-24
-1.000345496	8.23E-16	1.13E-14
-1.000384446	0.00039896	0.00123809
-1.00088539	0.00446173	0.01098211
-1.002023119	1.35E-06	6.47E-06
-1.00295465	1.22E-21	2.72E-20
-1.003086489	0.00348038	0.00878092
-1.003103685	0.00600854	0.01437614
-1.003426831	6.96E-06	2.96E-05
-1.003600407	7.71E-14	8.97E-13
-1.003654817	0.00101392	0.00287014
-1.003985512	2.13E-26	6.86E-25
-1.004077366	1.76E-20	3.57E-19
-1.004365691	7.93E-12	7.46E-11
-1.005853062	2.21E-21	4.83E-20
-1.006751993	1.36E-13	1.54E-12
-1.007670103	1.06E-05	4.41E-05
-1.009049702	8.33E-08	4.80E-07
-1.009380922	0.00559669	0.01350707
-1.009823953	0.00031341	0.00099435
-1.011305118	3.65E-27	1.25E-25
-1.012449896	0.00022692	0.00074084
-1.013621272	2.83E-11	2.51E-10

-1.013886273	0.00043449	0.00134071
-1.014417869	0.00183271	0.00493874
-1.015798758	1.34E-38	8.63E-37
-1.015987551	3.28E-25	9.57E-24
-1.016066841	6.39E-16	8.89E-15
-1.016435316	1.52E-11	1.39E-10
-1.016873833	0.00025697	0.00083059
-1.01748084	4.89E-06	2.14E-05
-1.019225963	9.67E-09	6.29E-08
-1.020269745	2.33E-22	5.56E-21
-1.020952464	5.61E-06	2.43E-05
-1.02192199	6.47E-12	6.15E-11
-1.021992955	3.08E-09	2.14E-08
-1.023049738	0.00501655	0.01222179
-1.023621583	5.06E-11	4.35E-10
-1.024716351	0.00040607	0.001259
-1.025466894	0.00315984	0.00806034
-1.025896495	0.00048239	0.00147479
-1.026171593	0.00175108	0.0047394
-1.026370709	1.05E-09	7.74E-09
-1.027289484	9.49E-13	9.87E-12
-1.027753033	8.86E-08	5.07E-07
-1.028243909	1.08E-06	5.27E-06
-1.02842131	1.93E-20	3.90E-19
-1.029349342	0.00479346	0.01172021
-1.029435366	0.00477478	0.01168294
-1.029821215	8.30E-06	3.50E-05
-1.031282396	3.16E-07	1.67E-06
-1.031499939	0.00015031	0.00050902
-1.033624387	4.00E-44	3.43E-42
-1.034688286	5.02E-05	0.0001863
-1.034727506	1.66E-21	3.67E-20
-1.036789288	7.82E-13	8.20E-12
-1.037984244	0.00033005	0.00104107
-1.038071314	0.00046531	0.0014261
-1.038219442	8.61E-06	3.62E-05
-1.038791135	7.08E-07	3.54E-06
-1.040556557	2.32E-25	6.88E-24
-1.040838788	0.00262505	0.00682012
-1.042058059	9.61E-11	8.01E-10
-1.042623199	1.62E-22	3.92E-21
-1.042774839	6.90E-08	4.02E-07
-1.042883182	1.18E-20	2.44E-19
-1.043152163	0.00394231	0.00983158

-1.043206333	1.17E-18	2.07E-17
-1.044342146	2.44E-17	3.84E-16
-1.045411885	4.92E-10	3.80E-09
-1.045639506	0.00082791	0.0023913
-1.04567483	1.21E-13	1.39E-12
-1.046308779	3.32E-05	0.00012741
-1.046908093	4.19E-12	4.06E-11
-1.047788046	3.59E-05	0.00013692
-1.047919522	7.46E-10	5.59E-09
-1.048007841	5.35E-05	0.00019764
-1.048411288	1.77E-30	7.44E-29
-1.0493371	0.00302542	0.00775671
-1.050844675	0.00013798	0.00047173
-1.051519643	4.72E-13	5.08E-12
-1.053003251	8.03E-10	5.99E-09
-1.057186221	4.78E-20	9.37E-19
-1.057853779	7.65E-05	0.00027451
-1.058646261	5.59E-05	0.00020571
-1.058854029	3.00E-05	0.00011588
-1.060390384	6.73E-06	2.87E-05
-1.064819064	0.00030192	0.00096186
-1.065136488	2.44E-05	9.59E-05
-1.066232981	4.75E-08	2.83E-07
-1.066589469	7.03E-09	4.66E-08
-1.067428938	1.34E-08	8.60E-08
-1.068061778	5.07E-36	2.88E-34
-1.068106582	1.98E-11	1.78E-10
-1.068224304	1.92E-22	4.60E-21
-1.068727147	0.00250289	0.00653642
-1.068759502	0.00338284	0.00856978
-1.069891532	3.28E-15	4.31E-14
-1.070788329	6.16E-17	9.42E-16
-1.071831498	0.00054618	0.00164871
-1.072899015	8.24E-24	2.18E-22
-1.073263855	0.00276652	0.00714539
-1.075659802	2.23E-08	1.39E-07
-1.07656642	2.05E-20	4.13E-19
-1.076581172	1.34E-20	2.75E-19
-1.079123401	7.95E-06	3.36E-05
-1.079778156	4.39E-08	2.62E-07
-1.080404635	9.37E-22	2.11E-20
-1.081675226	0.00021594	0.00070754
-1.082178002	3.69E-15	4.83E-14
-1.084599414	9.57E-26	2.92E-24

-1.08486855	4.13E-06	1.84E-05
-1.086382818	9.00E-15	1.15E-13
-1.086492851	4.04E-06	1.80E-05
-1.08680791	6.50E-22	1.48E-20
-1.089187927	5.30E-24	1.43E-22
-1.089777269	0.00012432	0.00042869
-1.091285002	0.00075729	0.00220955
-1.093246466	6.34E-06	2.72E-05
-1.093786647	1.78E-05	7.14E-05
-1.094198377	3.81E-17	5.89E-16
-1.094913166	4.62E-20	9.10E-19
-1.09527276	1.21E-05	4.98E-05
-1.096538543	2.42E-13	2.68E-12
-1.097070196	4.24E-07	2.20E-06
-1.097213812	6.74E-05	0.00024416
-1.098102238	4.87E-16	6.93E-15
-1.099124666	2.37E-10	1.90E-09
-1.099515801	0.00143099	0.00394443
-1.099885435	2.29E-12	2.28E-11
-1.100522053	0.00143008	0.00394272
-1.10194736	0.00138623	0.00383035
-1.103094458	1.16E-12	1.20E-11
-1.104070726	0.00159748	0.00436096
-1.104666224	9.52E-15	1.21E-13
-1.105520984	4.27E-14	5.07E-13
-1.10904997	7.28E-05	0.00026231
-1.10979464	0.00236273	0.00620372
-1.112503297	6.21E-11	5.29E-10
-1.113905266	3.20E-16	4.65E-15
-1.114433445	1.12E-15	1.52E-14
-1.114449297	4.29E-06	1.90E-05
-1.118640281	5.95E-13	6.34E-12
-1.118810095	4.39E-06	1.94E-05
-1.119723441	9.51E-16	1.30E-14
-1.120547157	1.73E-05	6.94E-05
-1.122182627	4.26E-47	4.43E-45
-1.124552311	4.21E-26	1.33E-24
-1.126584741	5.67E-53	7.56E-51
-1.128954096	2.10E-06	9.79E-06
-1.130715898	0.00018448	0.00061256
-1.131577161	2.48E-06	1.14E-05
-1.132336345	2.71E-22	6.43E-21
-1.133804388	5.46E-14	6.45E-13
-1.134298257	1.44E-05	5.86E-05

-1.135829134	4.98E-05	0.0001849
-1.136197741	1.30E-29	5.15E-28
-1.136953909	1.26E-13	1.44E-12
-1.138231461	1.37E-07	7.60E-07
-1.139184052	1.77E-10	1.44E-09
-1.141016568	3.00E-24	8.15E-23
-1.141237129	4.71E-11	4.07E-10
-1.146176192	3.00E-06	1.36E-05
-1.148610341	4.03E-20	7.99E-19
-1.1490637	4.04E-09	2.76E-08
-1.151124843	1.51E-13	1.71E-12
-1.151947849	0.00057645	0.00173127
-1.152102431	2.47E-06	1.14E-05
-1.153903627	1.47E-23	3.84E-22
-1.15539404	2.23E-51	2.71E-49
-1.156092147	2.20E-10	1.77E-09
-1.157545917	0.00031163	0.00098987
-1.157725343	4.86E-21	1.03E-19
-1.158231632	0.00133423	0.00369791
-1.160802644	6.69E-15	8.58E-14
-1.160827391	0.00144572	0.00398098
-1.163698614	1.06E-13	1.23E-12
-1.164885409	8.80E-07	4.35E-06
-1.166513583	9.22E-08	5.26E-07
-1.168354516	2.15E-08	1.35E-07
-1.174565156	8.64E-08	4.95E-07
-1.178270078	4.64E-13	4.99E-12
-1.179658306	1.79E-14	2.20E-13
-1.179703732	0.00053048	0.00160632
-1.18213876	0.00118515	0.00331647
-1.182177102	7.89E-12	7.42E-11
-1.184783673	4.54E-20	8.97E-19
-1.184805397	1.34E-07	7.45E-07
-1.185828468	1.28E-21	2.85E-20
-1.188403193	1.44E-07	7.96E-07
-1.189788434	1.49E-09	1.07E-08
-1.190627747	0.00030181	0.00096179
-1.195872591	6.74E-06	2.88E-05
-1.196760099	0.00090668	0.0025921
-1.19762713	0.0010333	0.00292095
-1.198728031	0.00013438	0.00046024
-1.200589038	0.00060398	0.00180557
-1.204238221	4.21E-07	2.18E-06
-1.205029526	2.97E-10	2.36E-09

-1.206660064	7.52E-17	1.14E-15
-1.210215073	0.00037148	0.00115967
-1.210355959	2.63E-11	2.33E-10
-1.210860469	1.14E-21	2.57E-20
-1.213455117	5.10E-09	3.45E-08
-1.214882282	1.45E-14	1.80E-13
-1.215907201	3.73E-10	2.93E-09
-1.216450301	0.00086397	0.00248249
-1.21651247	1.78E-41	1.29E-39
-1.218618771	1.86E-31	8.32E-30
-1.218811142	0.000182	0.00060491
-1.218985076	1.77E-13	1.99E-12
-1.219238631	3.50E-13	3.80E-12
-1.219292548	1.76E-26	5.75E-25
-1.219554865	5.70E-10	4.36E-09
-1.221349131	8.47E-19	1.52E-17
-1.225571833	8.46E-19	1.52E-17
-1.225761334	1.65E-08	1.04E-07
-1.226235684	4.11E-15	5.36E-14
-1.228044493	2.63E-10	2.10E-09
-1.231238553	6.14E-34	3.04E-32
-1.233035831	4.64E-20	9.12E-19
-1.23334568	1.31E-13	1.49E-12
-1.233499329	0.00039508	0.00122801
-1.233569877	3.11E-07	1.65E-06
-1.234751351	2.96E-32	1.36E-30
-1.235104438	2.48E-11	2.21E-10
-1.235801808	0.00031354	0.00099455
-1.236408112	1.84E-36	1.05E-34
-1.239704366	5.92E-35	3.15E-33
-1.242813312	4.18E-05	0.00015776
-1.242888493	6.28E-13	6.65E-12
-1.244789724	4.23E-26	1.33E-24
-1.246654918	6.43E-08	3.76E-07
-1.246816489	0.00014433	0.00049075
-1.247667351	3.92E-06	1.75E-05
-1.249810632	0.00058064	0.00174277
-1.254456283	0.00019825	0.00065445
-1.25485779	2.66E-16	3.88E-15
-1.255623728	2.50E-08	1.55E-07
-1.256791044	1.38E-36	7.96E-35
-1.256882424	2.01E-31	8.94E-30
-1.257020347	5.59E-10	4.28E-09
-1.257057854	2.20E-05	8.68E-05

-1.258695077	1.07E-05	4.44E-05
-1.260930148	6.37E-18	1.05E-16
-1.261287967	0.00052267	0.00158372
-1.263161377	2.24E-17	3.54E-16
-1.264901359	1.26E-14	1.57E-13
-1.26595115	5.03E-05	0.00018651
-1.270876621	2.26E-06	1.05E-05
-1.27220722	2.41E-15	3.20E-14
-1.27352812	4.14E-18	6.98E-17
-1.276485394	5.12E-06	2.24E-05
-1.279288175	3.65E-09	2.51E-08
-1.280805395	0.00037939	0.0011822
-1.283928595	3.46E-45	3.20E-43
-1.285400496	4.17E-12	4.05E-11
-1.28604961	8.95E-12	8.36E-11
-1.290668211	2.58E-11	2.30E-10
-1.291068508	0.00028318	0.00090755
-1.291152793	0.00034673	0.00109006
-1.295132719	1.10E-05	4.57E-05
-1.295749456	1.18E-09	8.58E-09
-1.296606946	6.89E-06	2.94E-05
-1.29864912	6.73E-10	5.09E-09
-1.299656695	0.00013001	0.00044617
-1.302300449	1.31E-06	6.32E-06
-1.305572684	2.83E-11	2.51E-10
-1.307657487	7.41E-05	0.00026667
-1.310531684	8.60E-08	4.93E-07
-1.31110634	1.01E-34	5.28E-33
-1.311475449	3.26E-08	1.99E-07
-1.325464484	2.07E-45	1.93E-43
-1.325956514	9.89E-06	4.13E-05
-1.326908956	2.49E-06	1.15E-05
-1.332738271	5.16E-16	7.31E-15
-1.335396927	2.34E-34	1.19E-32
-1.338380501	0.00015969	0.00053772
-1.338527772	8.35E-06	3.52E-05
-1.341155983	4.21E-09	2.87E-08
-1.343283944	1.61E-11	1.47E-10
-1.348455186	6.49E-29	2.46E-27
-1.359379479	1.63E-23	4.22E-22
-1.360333922	2.66E-17	4.17E-16
-1.362702311	4.97E-18	8.34E-17
-1.363361263	3.02E-17	4.70E-16
-1.368997569	7.03E-10	5.29E-09

-1.374185581	3.03E-10	2.40E-09
-1.381762494	0.00015182	0.00051357
-1.383314577	0.00014425	0.00049059
-1.387301135	1.42E-06	6.77E-06
-1.387429884	6.21E-33	2.91E-31
-1.39208825	2.10E-16	3.09E-15
-1.394006311	1.73E-05	6.96E-05
-1.400572757	2.00E-11	1.80E-10
-1.402265441	1.41E-10	1.16E-09
-1.402409759	2.67E-14	3.25E-13
-1.404457082	0.00010142	0.0003555
-1.408750359	7.79E-50	8.99E-48
-1.414885194	3.46E-09	2.38E-08
-1.428727661	5.57E-26	1.73E-24
-1.432474078	3.33E-26	1.06E-24
-1.443008114	2.18E-06	1.02E-05
-1.445380872	2.72E-35	1.48E-33
-1.447535727	4.91E-29	1.87E-27
-1.448402631	9.76E-16	1.33E-14
-1.45876427	6.97E-19	1.26E-17
-1.461074673	7.44E-21	1.56E-19
-1.461573615	1.12E-10	9.29E-10
-1.462154401	7.61E-08	4.41E-07
-1.46575488	1.38E-11	1.27E-10
-1.475208821	3.84E-11	3.35E-10
-1.475786489	3.67E-09	2.52E-08
-1.483268826	2.14E-06	9.97E-06
-1.489097064	1.59E-11	1.45E-10
-1.491399857	6.07E-15	7.81E-14
-1.492040487	7.03E-17	1.07E-15
-1.49230336	2.68E-27	9.27E-26
-1.498363559	1.04E-30	4.45E-29
-1.501228267	1.08E-13	1.25E-12
-1.501890729	2.71E-23	6.97E-22
-1.508291194	2.76E-15	3.64E-14
-1.508936325	2.25E-37	1.37E-35
-1.512072797	8.72E-20	1.67E-18
-1.512749447	3.37E-13	3.67E-12
-1.520452906	1.79E-10	1.45E-09
-1.524886858	1.12E-17	1.82E-16
-1.542388984	8.72E-10	6.49E-09
-1.5450674	1.21E-09	8.81E-09
-1.553623332	1.41E-06	6.72E-06
-1.565152316	1.11E-06	5.39E-06

-1.571316422	1.30E-23	3.40E-22
-1.573005624	1.44E-16	2.15E-15
-1.576801379	1.04E-12	1.08E-11
-1.580709004	1.17E-10	9.69E-10
-1.581215729	6.07E-13	6.45E-12
-1.58481335	1.28E-06	6.17E-06
-1.591807687	3.48E-19	6.36E-18
-1.595556984	1.27E-14	1.58E-13
-1.612996738	2.00E-06	9.36E-06
-1.614327174	5.96E-09	3.99E-08
-1.623531106	2.26E-09	1.59E-08
-1.628248832	1.78E-18	3.11E-17
-1.628279658	1.62E-33	7.75E-32
-1.634374155	2.45E-31	1.08E-29
-1.639001288	8.87E-08	5.07E-07
-1.644736093	4.77E-60	9.69E-58
-1.645512955	4.25E-09	2.90E-08
-1.647306376	8.10E-23	2.00E-21
-1.650086193	8.41E-11	7.08E-10
-1.651324951	8.17E-23	2.02E-21
-1.674640643	3.51E-08	2.13E-07
-1.683228734	7.38E-08	4.28E-07
-1.695018545	3.09E-07	1.64E-06
-1.696285168	4.22E-18	7.12E-17
-1.702301354	1.50E-26	4.95E-25
-1.705854608	8.61E-13	8.99E-12
-1.707249325	5.68E-16	7.96E-15
-1.707767465	2.91E-08	1.78E-07
-1.708164355	1.63E-12	1.66E-11
-1.713823006	2.76E-25	8.10E-24
-1.71828461	1.13E-10	9.34E-10
-1.721725336	1.58E-06	7.49E-06
-1.725723989	4.76E-20	9.34E-19
-1.726870842	1.27E-08	8.13E-08
-1.72810983	8.49E-13	8.87E-12
-1.732308716	1.79E-17	2.83E-16
-1.733983799	9.32E-25	2.62E-23
-1.741624511	1.06E-08	6.90E-08
-1.766655109	7.54E-10	5.65E-09
-1.793826324	2.26E-09	1.59E-08
-1.832166147	1.25E-30	5.30E-29
-1.888039763	1.21E-19	2.30E-18
-1.902838761	1.03E-23	2.70E-22
-1.918453604	2.88E-11	2.54E-10

-1.941198357	9.87E-32	4.46E-30
-1.947799877	3.95E-14	4.72E-13
-1.959999105	6.18E-19	1.12E-17
-1.967045761	3.81E-41	2.75E-39
-1.974243162	1.06E-10	8.77E-10
-1.974733862	2.22E-11	1.99E-10
-2.017639557	1.01E-31	4.56E-30
-2.039086819	3.77E-12	3.68E-11
-2.067824888	3.94E-15	5.15E-14
-2.095327498	6.88E-22	1.56E-20
-2.146443546	1.56E-17	2.49E-16
-2.152487163	1.35E-63	3.23E-61
-2.222803598	9.20E-46	8.89E-44
-2.251322764	1.17E-25	3.54E-24
-2.268510493	3.33E-58	5.96E-56
-2.380266094	2.03E-26	6.57E-25
-2.82840585	1.44E-28	5.37E-27
