# Supplementary material for: HGF/MET Axis Induces Tumor Secretion of Tenascin-C and Promotes Stromal Rewiring in Pancreatic Cancer
Source: Cancers (Basel). 2021 Jul 14;13(14):3519. doi: 10.3390/cancers13143519 (PMC8305254; doi:10.3390/cancers13143519)
Supplement: Supplementary file 1 [file cancers-13-03519-s001.zip › Table S2_Modica et al..pdf]

Suppl. Table S2: differentially expressed genes (untreated vs HGF+Co

symbol	ensembl gene ID	baseMean	log2FoldChange
PRR15L	ENSG00000167183	418.4757737	1.609846214
SLC40A1	ENSG00000138449	692.9929291	1.510285231
B4GAT1	ENSG00000174684	172.7153046	1.489967926
RBBP8NL	ENSG00000130701	46.20802283	1.465435618
PLCH1	ENSG00000114805	284.9739725	1.357507902
CLDN2	ENSG00000165376	222.7719504	1.343076556
IKZF2	ENSG00000030419	92.95180959	1.340686848
ISX	ENSG00000175329	381.7710615	1.322238097
C2orf72	ENSG00000204128	77.22669135	1.304836985
GRAMD1C	ENSG00000178075	204.5508011	1.262202653
TNFSF10	ENSG00000121858	321.3831693	1.222519025
C2orf88	ENSG00000187699	261.6839322	1.209346602
TMEM37	ENSG00000171227	206.6429645	1.201988438
COLCA2	ENSG00000214290	72.50779605	1.188817194
LRRC66	ENSG00000188993	165.0579814	1.187006898
EPN3	ENSG00000049283	202.8595976	1.16969869
TRIM2	ENSG00000109654	750.3002229	1.166106984
PDK4	ENSG00000004799	114.5399158	1.1606563
ACSS1	ENSG00000154930	157.6750987	1.158336468
E2F2	ENSG00000007968	124.1403034	1.154408952
RASL11A	ENSG00000122035	353.3590716	1.149167848
ATP10B	ENSG00000118322	1335.06413	1.141889658
LRRC31	ENSG00000114248	144.7281745	1.136851724
FFAR4	ENSG00000186188	148.327134	1.116273255
SMPD3	ENSG00000103056	69.22783452	1.101473797
MEF2C	ENSG00000081189	234.2925565	1.099306644
PKDCC	ENSG00000162878	2001.592849	1.08350001
RHOU	ENSG00000116574	467.6288523	1.076489199
PBX1	ENSG00000185630	111.916253	1.066938383
SESN3	ENSG00000149212	294.6919034	1.060044064
FRAT1	ENSG00000165879	38.25809161	1.047500444
AGFG2	ENSG00000106351	518.7940356	1.046845872
KCNE3	ENSG00000175538	387.1241024	1.024600682
KLHDC7A	ENSG00000179023	32.65630176	1.022555174
CDX2	ENSG00000165556	1299.160212	1.021684129
SOWAHA	ENSG00000198944	64.13398424	1.020116523
KIAA1211L	ENSG00000196872	91.16941998	1.017445783
ICK	ENSG00000112144	117.5429483	1.015434675
GNG2	ENSG00000186469	98.9326669	1.00711516
RAB40B	ENSG00000141542	92.47772673	1.002534406
ARHGAP29	ENSG00000137962	4159.892549	-1.002822583

DKK1	ENSG00000107984	15171.63575	-1.011703273
F3	ENSG00000117525	31391.37993	-1.013352978
ODC1	ENSG00000115758	7886.172253	-1.019574153
RASAL2	ENSG00000075391	1239.930027	-1.022730174
DUSP5	ENSG00000138166	3220.617603	-1.025652239
LAMA3	ENSG00000053747	7261.137201	-1.027883169
PLAU	ENSG00000122861	6869.405856	-1.031675613
FRMD6	ENSG00000139926	649.0781898	-1.035148854
MDFI	ENSG00000112559	786.8246881	-1.043050477
ALOXE3	ENSG00000179148	110.0364911	-1.047621321
MARCH_4	ENSG00000144583	24.65835871	-1.048651102
PMEPA1	ENSG00000124225	3442.608454	-1.056240164
BMP6	ENSG00000153162	43.54475506	-1.058199633
TUBB6	ENSG00000176014	1857.842864	-1.059039621
ABL2	ENSG00000143322	466.2317344	-1.062927245
PIK3CD	ENSG00000171608	31.62521589	-1.072807478
CXCL8	ENSG00000169429	279.9904945	-1.078501804
TGM2	ENSG00000198959	1480.780227	-1.087322295
IFFO2	ENSG00000169991	1369.963584	-1.087899588
FGFBP1	ENSG00000137440	1026.062419	-1.099251229
CLCF1	ENSG00000175505	618.9560221	-1.102696444
SOCS2	ENSG00000120833	164.4005854	-1.104980413
PHLDB2	ENSG00000144824	316.5262511	-1.114324071
TGFA	ENSG00000163235	5748.737574	-1.115945462
KIAA1462	ENSG00000165757	133.7254999	-1.127867544
KLF7	ENSG00000118263	775.3400907	-1.130636938
SLC9A2	ENSG00000115616	274.0065523	-1.131299364
TMEM158	ENSG00000249992	95.55676281	-1.131565934
CD274	ENSG00000120217	227.7036227	-1.146815887
MMP1	ENSG00000196611	9597.236413	-1.154091446
KRT17	ENSG00000128422	21.85298551	-1.165565047
PORCN	ENSG00000102312	832.5467692	-1.201185754
SPRR3	ENSG00000163209	58.5121535	-1.202413406
GLIPR1	ENSG00000139278	709.0685729	-1.20463722
LRRC8C	ENSG00000171488	84.76807441	-1.22192445
CHST11	ENSG00000171310	1267.059559	-1.225054633
BCAR3	ENSG00000137936	1545.553546	-1.230540749
KIAA1549L	ENSG00000110427	552.9060622	-1.237230945
S100A2	ENSG00000196754	167.1993957	-1.242183953
TM4SF19	ENSG00000145107	148.4628072	-1.245524108
TNC	ENSG00000041982	544.9848471	-1.254286441
KRTAP3-1	ENSG00000212901	51.38081958	-1.25445332
SH2D5	ENSG00000189410	158.0169734	-1.285090456
FGFR1	ENSG00000077782	898.9278649	-1.285699185

GFOD1	ENSG00000145990	458.8753928	-1.300118377
FAM83A	ENSG00000147689	3221.860808	-1.302088379
MDGA1	ENSG00000112139	202.6177554	-1.307218897
CGB8	ENSG00000213030	64.59079669	-1.309260446
PTRF	ENSG00000177469	615.2107113	-1.313879566
ZBED2	ENSG00000177494	1708.815764	-1.316655288
ARID3B	ENSG00000179361	374.1944238	-1.318400584
THBD	ENSG00000178726	3147.182712	-1.333220352
OSBP2	ENSG00000184792	502.7382456	-1.333838615
CPA4	ENSG00000128510	238.4407832	-1.334628538
TMCC3	ENSG00000057704	806.0921596	-1.353079017
WNT7A	ENSG00000154764	93.33127697	-1.367665959
LAMB3	ENSG00000196878	8738.385373	-1.367917287
FERMT2	ENSG00000073712	186.7506369	-1.375798866
TBC1D2	ENSG00000095383	824.8424885	-1.399199262
ITGA5	ENSG00000161638	211.3608059	-1.403808913
NRIP3	ENSG00000175352	149.8894582	-1.488227949
ADRB2	ENSG00000169252	192.3265752	-1.512332579
LAMC2	ENSG00000058085	19875.15748	-1.513062265
ETS1	ENSG00000134954	4493.533137	-1.518545097
PEAR1	ENSG00000187800	532.6728506	-1.526505895
SERPINE1	ENSG00000106366	316.2975973	-1.561329456
SNAI2	ENSG00000019549	774.6855218	-1.56404429
LOXL2	ENSG00000134013	217.8836515	-1.58321798
SPOCD1	ENSG00000134668	211.2222505	-1.588378715
MMP10	ENSG00000166670	512.2692024	-1.607883562
UPP1	ENSG00000183696	1410.991458	-1.613911537
TNFAIP3	ENSG00000118503	444.4287567	-1.64244534
AKAP12	ENSG00000131016	10402.08071	-1.647588105
DCLK1	ENSG00000133083	94.23404246	-1.678602789
FOSL1	ENSG00000175592	4016.00735	-1.719778274
NAV3	ENSG00000067798	150.0459026	-1.839973443
RELN	ENSG00000189056	125.5109664	-1.941025512
CGB5	ENSG00000189052	289.5435591	-2.012204062
SPRR1B	ENSG00000169469	81.63056767	-2.021882409
IL1RL1	ENSG00000115602	128.2220729	-2.083587734
SEMA7A	ENSG00000138623	714.4600124	-2.240894635
G0S2	ENSG00000123689	420.7026442	-2.358423864
SERPINB2	ENSG00000197632	38.14757941	-2.456307183
KRT6A	ENSG00000205420	276.7473929	-2.661880827

mbo-treated HPAF-II cells)

pvalue	padj
2.12E-33	4.04E-31
1.09E-49	4.88E-47
9.36E-25	9.09E-23
5.43E-12	1.56E-10
3.83E-28	5.18E-26
2.50E-22	1.99E-20
4.87E-12	1.42E-10
4.56E-21	3.34E-19
1.03E-12	3.25E-11
6.68E-19	4.34E-17
9.80E-17	4.93E-15
1.31E-15	5.87E-14
7.86E-16	3.62E-14
6.87E-10	1.41E-08
6.10E-16	2.85E-14
5.82E-18	3.30E-16
4.56E-23	3.80E-21
1.19E-10	2.73E-09
1.88E-15	8.31E-14
1.22E-11	3.35E-10
5.87E-22	4.56E-20
3.72E-30	5.93E-28
8.99E-11	2.14E-09
8.49E-15	3.50E-13
7.23E-09	1.28E-07
3.40E-13	1.15E-11
3.78E-35	8.66E-33
3.16E-20	2.20E-18
2.55E-10	5.60E-09
8.54E-14	3.13E-12
1.28E-06	1.43E-05
6.63E-27	7.94E-25
1.49E-06	1.63E-05
2.99E-06	3.07E-05
9.09E-28	1.17E-25
6.09E-08	9.08E-07
4.64E-09	8.48E-08
9.86E-10	1.96E-08
7.74E-10	1.57E-08
1.15E-08	1.95E-07
1.63E-34	3.36E-32

2.66E-47	1.08E-44
8.78E-61	7.89E-58
5.76E-62	6.04E-59
3.82E-30	6.01E-28
7.83E-38	2.05E-35
3.65E-40	1.09E-37
7.25E-36	1.75E-33
1.68E-26	1.94E-24
3.18E-29	4.71E-27
1.13E-10	2.62E-09
4.39E-06	4.32E-05
2.92E-50	1.41E-47
4.65E-07	5.76E-06
1.92E-50	9.67E-48
1.12E-21	8.41E-20
1.75E-06	1.89E-05
4.64E-17	2.43E-15
1.52E-27	1.91E-25
5.91E-50	2.75E-47
7.04E-31	1.18E-28
8.96E-27	1.04E-24
3.56E-14	1.35E-12
3.28E-21	2.43E-19
7.62E-55	4.85E-52
3.78E-10	8.10E-09
1.24E-25	1.34E-23
6.59E-17	3.38E-15
1.17E-11	3.20E-10
5.29E-17	2.74E-15
4.82E-46	1.90E-43
4.59E-07	5.70E-06
3.67E-32	6.41E-30
1.14E-09	2.25E-08
2.57E-30	4.15E-28
3.36E-12	1.01E-10
2.83E-51	1.48E-48
5.37E-67	6.76E-64
9.25E-33	1.66E-30
1.91E-18	1.16E-16
1.81E-15	8.06E-14
2.62E-39	7.50E-37
4.26E-10	8.98E-09
4.55E-19	2.97E-17
3.62E-44	1.30E-41

3.17E-26	3.57E-24
2.99E-76	7.53E-73
6.50E-22	5.02E-20
1.93E-11	5.12E-10
2.17E-47	9.11E-45
7.46E-73	1.34E-69
7.50E-28	9.83E-26
5.53E-52	3.02E-49
1.49E-40	4.57E-38
2.95E-18	1.73E-16
4.21E-55	2.94E-52
1.84E-14	7.29E-13
3.08E-63	3.52E-60
1.49E-20	1.08E-18
4.60E-40	1.35E-37
3.40E-23	2.87E-21
1.09E-23	9.77E-22
2.55E-20	1.79E-18
1.25E-111	5.24E-108
9.02E-111	2.84E-107
1.94E-44	7.18E-42
3.16E-28	4.33E-26
2.67E-55	1.98E-52
3.15E-29	4.71E-27
9.57E-29	1.40E-26
5.35E-43	1.77E-40
2.34E-61	2.26E-58
3.03E-36	7.64E-34
1.74E-70	2.73E-67
1.46E-18	8.94E-17
1.76E-127	2.21E-123
1.73E-29	2.65E-27
6.77E-25	6.71E-23
9.42E-61	7.90E-58
2.97E-27	3.67E-25
2.38E-30	3.89E-28
4.15E-112	2.61E-108
3.87E-73	8.13E-70
4.00E-27	4.89E-25
3.40E-67	4.75E-64
