# Supplementary material for: HGF/MET Axis Induces Tumor Secretion of Tenascin-C and Promotes Stromal Rewiring in Pancreatic Cancer
Source: Cancers (Basel). 2021 Jul 14;13(14):3519. doi: 10.3390/cancers13143519 (PMC8305254; doi:10.3390/cancers13143519)
Supplement: Supplementary file 1 [file cancers-13-03519-s001.zip › Table S3_Modica et al..pdf]

Suppl. Table S3: IPA analysis of differentially expressed genes (untreated vs HGF+Combo-treated HPAF-II cells)

Categories	Diseases or Functions Annotation	p-Value	Predicted Activation State	Activation z-score	Molecules	# Molecules
Cellular Movement	Invasion of cells	5.18E-14	Decreased	-4,059	ABL2,ADRB2,AKA	35
Cellular Movement	Cell movement	8.44E-13	Decreased	-3,080	ABL2,ADRB2,AKA	53
Cardiovascular System D	Angiogenesis	3.32E-11	Decreased	-2,408	ADRB2,BMP6,CD	31
Cardiovascular System D	Development of vasculature	3.66E-11	Decreased	-2,408	ABL2,ADRB2,ARI	33
Cellular Movement	Migration of cells	4.02E-11	Decreased	-3,240	ABL2,ADRB2,AKA	47
Cancer,Organismal Injury	Growth of tumor	1.06E-10	Decreased	-2,349	BMP6,CD274,CGI	29
Cellular Movement	Cell movement of tumor cell lines	4.39E-10	Decreased	-2,720	ADRB2,AKAP12,B	30
Cellular Movement	Migration of tumor cell lines	3.21E-09	Decreased	-3,511	ADRB2,AKAP12,B	26
Cardiovascular System D	Vasculogenesis	3.95E-09	Decreased	-2,919	BMP6,CXCL8,DKK	25
Cancer,Organismal Injury	Progression of tumor	1.07E-08	Decreased	-2,236	ABL2,CD274,CXCI	15
Cardiovascular System D	Cell movement of endothelial cells	3.43E-08	Decreased	-2,090	BMP6,CXCL8,DKK	16
Cellular Movement	Invasion of tumor cell lines	7.07E-08	Decreased	-3,478	ABL2,ADRB2,AKA	23
Cancer,Organismal Injury	Invasion of tumor	8.17E-08	Decreased	-3,236	CD274,CDX2,ETS	12
Connective Tissue Disord	Damage of cartilage tissue	1.06E-07	Decreased	-2,433	CXCL8,DKK1,FGF	8
Cancer,Organismal Injury	Cancer of cells	1.23E-07	Decreased	-2,352	ABL2,ADRB2,AKA	64
Tissue Morphology	Quantity of cells	1.76E-07	Decreased	-2,402	ABL2,ADRB2,BCA	37
Cancer,Organismal Injury	Epithelial neoplasm	1.92E-07	Decreased	-2,027	ABL2,ACSS1,ADR	114
Cancer,Organismal Injury	Nonhematologic malignant neoplasm	2.31E-07	Decreased	-3,038	ABL2,ACSS1,ADR	116
Cardiovascular System D	Migration of endothelial cells	4.40E-07	Decreased	-2,103	BMP6,CXCL8,DKK	14
Cellular Movement	Invasion of breast cancer cell lines	5.00E-07	Decreased	-2,278	ABL2,ADRB2,BCA	13
Cellular Movement	Migration of tumor cells	5.04E-07	Decreased	-2,767	CXCL8,F3,ITGA5,	11
Cancer,Organismal Injury	Invasion of malignant tumor	5.38E-07	Decreased	-2,433	CDX2,ETS1,F3,FE	9
Cancer,Organismal Injury	Carcinoma	5.93E-07	Decreased	-2,753	ABL2,ACSS1,ADR	113
Cancer,Organismal Injury	Non-melanoma solid tumor	7.18E-07	Decreased	-2,303	ABL2,ACSS1,ADR	114
Cancer,Organismal Injury	Metastasis of cells	8.33E-07	Decreased	-2,137	CD274,CXCL8,F3,	13
Cellular Movement	Migration of cancer cells	9.28E-07	Decreased	-2,593	CXCL8,F3,ITGA5,	10
Cellular Movement	Migration of breast cancer cell lines	1.82E-06	Decreased	-2,635	ADRB2,BCAR3,BN	12
Cellular Movement	Cell movement of breast cancer cell lines	1.86E-06	Decreased	-2,697	ADRB2,BCAR3,BN	13
Cell Death and Survival	Cell survival	4.79E-06	Decreased	-2,921	ABL2,BMP6,CXCL	30
Inflammatory Response	Inflammatory response	5.72E-06	Decreased	-3,102	ADRB2,CLCF1,CX	20
Connective Tissue Disord	Abnormality of cartilage tissue	6.14E-06	Decreased	-2,433	ADRB2,CXCL8,DK	9
Cellular Movement	Cell movement of connective tissue cells	6.32E-06	Decreased	-2,339	ABL2,CXCL8,FGF	10
Cancer,Organismal Injury	Growth of carcinoma	6.42E-06	Decreased	-2,599	BMP6,CD274,DK	8

Connective Tissue Disord	Deterioration of connective tissue	6.82E-06	Decreased	-2,433	ADRB2,CXCL8,DK	9
Cancer,Organismal Injury	Growth of malignant tumor	9.06E-06	Decreased	-2,804	BMP6,CD274,CX(	14
Cancer,Cellular Developr	Proliferation of tumor cells	1.27E-05	Decreased	-2,482	CD274,CXCL8,DKI	14
Tissue Development	Growth of epithelial tissue	1.31E-05	Decreased	-2,213	BMP6,CXCL8,DKK	18
Cellular Movement	Cell movement of carcinoma cell lines	1.35E-05	Decreased	-3,077	CXCL8,ETS1,FERN	10
Cancer,Cellular Moveme	Invasion of tumor cells	2.17E-05	Decreased	-2,804	CDX2,F3,FERMT2	8
Cell-To-Cell Signaling and	Attachment of cells	3.16E-05	Decreased	-2,449	ETS1,F3,FERMT2,	7
Cell Death and Survival	Cell viability	3.81E-05	Decreased	-2,506	ABL2,BMP6,CXCL	27
Hematological System De	Accumulation of myeloid cells	3.82E-05	Decreased	-2,187	CXCL8,F3,FGFR1,I	8
Tissue Development	Accumulation of cells	4.02E-05	Decreased	-2,060	CD274,CXCL8,F3,	11
Cardiovascular System D	Vascularization	5.24E-05	Decreased	-2,110	CGB3 (includes o	9
Cancer,Cell Death and Su	Cell death of tumor	6.43E-05	Increased	2,262	BMP6,CXCL8,E2F	13
Hematological System De	Accumulation of phagocytes	7.19E-05	Decreased	-2,187	CXCL8,F3,FGFR1,I	7
Cellular Assembly and Or	Organization of cytoskeleton	1.06E-04	Decreased	-3,013	ABL2,AKAP12,B4(	26
Cellular Growth and Prol	Outgrowth of cells	1.26E-04	Decreased	-2,579	BMP6,CD274,FGI	12
Cancer,Organismal Injury	Metastasis of tumor cell lines	1.44E-04	Decreased	-2,121	CD274,CXCL8,F3,	9
Cellular Movement	Migration of carcinoma cell lines	1.48E-04	Decreased	-2,763	CXCL8,ETS1,ITGA	8
Cellular Growth and Prol	Proliferation of epithelial cells	1.48E-04	Decreased	-2,244	BMP6,CXCL8,DKK	13
Cellular Development,Ce	Proliferation of muscle cells	1.50E-04	Decreased	-2,028	CXCL8,F3,ITGA5,(	11
Cellular Development,Ce	Cell proliferation of carcinoma cell lines	1.68E-04	Decreased	-2,191	DKK1,F3,FGFR1,F	12
Cancer,Cell Death and Su	Cell death of tumor cells	2.01E-04	Increased	2,262	BMP6,CXCL8,E2F	12
Cancer,Cell Death and Su	Apoptosis of tumor cells	2.37E-04	Increased	2,361	E2F2,ETS1,LAMA:	9
Cellular Development,Ce	Outgrowth of neurites	2.41E-04	Decreased	-2,410	BMP6,FGFR1,KLF	11
Cellular Development,Ce	Growth of neurites	3.63E-04	Decreased	-2,738	BMP6,DCLK1,FGF	12
Cellular Movement	Invasion of carcinoma cell lines	3.90E-04	Decreased	-2,768	DKK1,FGFR1,FOSI	8
Cellular Movement	Cell movement of lung cancer cell lines	4.03E-04	Decreased	-2,378	ETS1,FOSL1,ITGA	6
Nervous System Develop	Quantity of neurons	4.26E-04	Decreased	-2,564	ABL2,ADRB2,BCA	11
Cell-To-Cell Signaling and	Recruitment of phagocytes	4.99E-04	Decreased	-2,043	CD274,CXCL8,ETS	8
Cell Morphology,Cellular	Formation of cellular protrusions	6.41E-04	Decreased	-2,883	ABL2,AKAP12,B4(	18
Lipid Metabolism,Small M	Synthesis of lipid	6.43E-04	Decreased	-2,292	ACSS1,ADRB2,AL(	16
Hematological System De	Quantity of macrophages	7.01E-04	Increased	2,216	F3,IL1RL1,PLAU,S	7
