# Supplementary material for: HGF/MET Axis Induces Tumor Secretion of Tenascin-C and Promotes Stromal Rewiring in Pancreatic Cancer
Source: Cancers (Basel). 2021 Jul 14;13(14):3519. doi: 10.3390/cancers13143519 (PMC8305254; doi:10.3390/cancers13143519)
Supplement: Supplementary file 1 [file cancers-13-03519-s001.zip › Table S4_Modica et al..pdf]

Supplementary Table S4: candidate genes mediating the tumor/stroma crosstalk

ACRONYM	NAME	FUNCTION	EXPRESSION PATTERN	ROLE IN DISEASE
CLCF1	Cardiotrophin like cytokine factor 1	Neurotrophic factor, B-cell stimulatory agent and neuroendocrine modulator of pituitary corticotroph function	.	Involved in cold-induced sweating syndrome 2 (CISS2)
MMP10	Matrix Metallo Peptidase 10	Matrix metalloproteinase; breaks down fibronectin, laminin, elastin, proteoglycan core protein, gelatins, and several types of collagen	Not expressed in normal tissues . Expression is induced in response to injury, infection, or transformation	Involved in arthritis and metastasis
LAMC2	Laminin Subunit Gamma 2	Subunit of laminin 5 (earlier known as kalinin); involved in the connection of epithelial cells to the basement membrane.	Expressed in several fetal tissues, specifically localized to epithelial cells in skin, lung and kidney	Associated with junctional epidermolysis bullosa
MMP1	Matrix Metallo Peptidase 1	Matrix metalloproteinase; cleaves several types of collagen	Esxpressed mainly in vascular tisssues; produced mostly by platelets	Associated with chronic obstructive pulmonary disease (COPD)
TGF A	Transforming growth factor alpha	Growth factor, ligand for EGF receptor; involved in cell proliferation, differentiation and development	Widely expressed in normal tissues	Associated with many types of cancers; involved in cleft lip/palate
PLAU	Plasminogen activator	Serine protease; converts the zymogen plasminogen to the active enzyme plasmin	Expressaed in a variety of tissues; upregulated in response to inflammatory cytokines	Associated with Quebec platelet disorder and late-onset Alzheimer's disease
SERPIN E1	Serpin family E member 1	Serine proteinase inhibitor; principal inhibitor of tissue plasminogen activator (tPA) and urokinase (uPA); inhibitor of fibrinolysis; involved in the regulation of cell adhesion and spreading	Widely expressed in various tissues; produced mainly by endothelial cells	Associated with plasminogen activator inhibitor-1 deficiency (PAI-1 deficiency) and thrombophilia
SERPIN B2	Serpin family B member 2	Serine proteinase inhibitor; inhibits urokinase-type plasminogen activator	Expression restricted to some cell types (keratinocytes, trophoblasts, macrophages, neurons), and is induced in response to cellular stress; produced mainly by monocytes	Associated with inflammatory diseases like pre-eclampsia, lupus, asthma, scleroderma, and periodontitis
TNC	Tenascin C	Extracellular matrix protein; modulator of cell adhesion; involved in neuron and axon guidance	Widely expressed in embryonic tissues; in adults, expression is very restricted, and is rapidly induced during wound healing, infection, inflammation , cancer; secreted mainly by stromal cells	In tumors, stimulates angiogenesis by elongation, migration and sprouting of endothelial cells
CXCL8	C-X-C Motif Chemokine Ligand 8	Chemotactic factor; major mediator of the inflammatory response; activates MMPs	Barely detectable in physiological conditions; expression is induced by pro-inflammatory cytokines; secreted mainly by neutrophils	Associated to the pathogenesis of bronchiolitis
